# Active layer dynamics drives a transition to biofilm fingering

**DOI:** 10.1038/s41522-023-00380-w

**Published:** 2023-04-06

**Authors:** Ellen Young, Gavin Melaugh, Rosalind J. Allen

**Affiliations:** 1 School of Physics and Astronomy, University of Edinburgh, Peter Guthrie Tait Road, Edinburgh, EH9 3FD United Kingdom; 2 Theoretical Microbial Ecology, Institute of Microbiology, Faculty of Biological Sciences, Friedrich Schiller University Jena, Buchaer Strasse 6, 07745 Jena, Germany

**Keywords:** Biofilms, Microbial ecology

## Abstract

The emergence of spatial organisation in biofilm growth is one of the most fundamental topics in biofilm biophysics and microbiology. It has long been known that growing biofilms can adopt smooth or rough interface morphologies, depending on the balance between nutrient supply and microbial growth; this ‘fingering’ transition has been linked with the average width of the ‘active layer’ of growing cells at the biofilm interface. Here we use long-time individual-based simulations of growing biofilms to investigate in detail the driving factors behind the biofilm-fingering transition. We show that the transition is associated with dynamical changes in the active layer. Fingering happens when gaps form in the active layer, which can cause local parts of the biofilm interface to pin, or become stationary relative to the moving front. Pinning can be transient or permanent, leading to different biofilm morphologies. By constructing a phase diagram for the transition, we show that the controlling factor is the magnitude of the relative fluctuations in the active layer thickness, rather than the active layer thickness per se. Taken together, our work suggests a central role for active layer dynamics in controlling the pinning of the biofilm interface and hence biofilm morphology.

## Introduction

Biofilms are diverse in their morphology. Biofilms grown under flow can be smooth or rough, or even ‘mushroom-shaped’^1–3^, while biofilms on liquid interfaces can show intricate wrinkly patterns^4^. Characterising distinct types of biofilm spatial structure, and the mechanisms by which they emerge, can lead to a better understanding of the underlying principles of this multicellular assembly process. It is also a prerequisite for understanding phenomena including genetic mixing and hence potential for cooperation, the extent of pathogen adhesion, as well as antibiotic penetration and the chances of fixation of antibiotic-resistant mutants^5–9^.

Biofilm spatial structure is often characterised in terms of the interface roughness, i.e. the standard deviation of the biofilm height. From a mechanistic point of view, it is well established that the interplay between local growth and the nutrient concentration field is important in controlling interface roughness^10–13^. Dockery and Klapper^11^ showed the existence of a fingering instability in which a local ‘bump’ on the growing interface tends to grow larger, since microbes in the ‘bump’ have better access to nutrients (diffusing from above) than those in adjacent areas; the growing bump then depletes nutrients from adjacent regions of the interface, further enhancing its growth. However, growth-generated pressure within the biofilm tends to fill in troughs in the biofilm interface, counteracting the tendency towards fingering^11,14^.

The balance between nutrient supply and microbial growth clearly lies at the heart of the biofilm-fingering transition. Analysis of the reaction-diffusion equation for nutrient close to the growing biofilm led Dockery and Klapper to identify a dimensionless controlling parameter ${({D}_{B}Y({k}_{S}+{S}_{{\mathrm{bulk}}})/({L}_{y}^{2}\rho {\mu }_{{\mathrm{max}}}))}^{\frac{1}{2}}$, which describes the balance between nutrient transport and microbial growth, and is associated with the distance that nutrient penetrates into the biofilm ^11^. Here, *L*_*y*_ is the horizontal system size, *Y* is the yield (units of biomass produced per unit of nutrient consumed), *k*_*S*_ the nutrient concentration for half-maximal growth, *S*_bulk_ is the nutrient concentration far from the biofilm, *D*_*B*_ is the diffusion constant of nutrient within the biofilm and *ρ* is the biomass density within the biofilm. In earlier work using a cellular automaton model, Picioreanu et al.^13^ had identified a similar parameter, but with *S*_bulk_ in place of the factor (*k*_*S*_ + *S*_bulk_) (and the system height in place of the lateral width). Nadell et al.^15^ also proposed a related parameter combination with units of distance, the ‘active layer depth’, to describe the thickness of the layer of growing cells at the top of the biofilm.

**Fig. 1:**
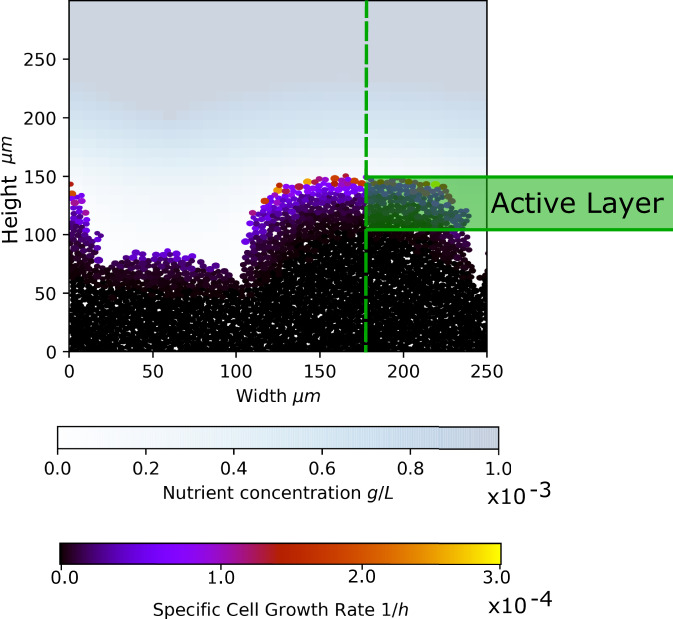
The concept of the active layer. A biofilm configuration generated in our simulations is shown, with the cells in the biofilm colour-coded according to their specific growth rate. The nutrient concentration field is shown on the blue scale. The nutrient is consumed by cells at the top of the biofilm, so that cells deeper in the biofilm are deprived of nutrients and do not proliferate. The active layer is defined as the layer of growing cells at the top of the biofilm (see Methods for further details).

While the combined parameters identified in these works are different, they all express the idea that the extent of nutrient penetration into the biofilm, which depends on the balance between nutrient supply and growth, is central in controlling spatial structure. Growth occurs only in this ‘active layer’ close to the biofilm interface that has access to nutrients, while cells deeper within the biofilm are not able to grow^15–19^ (Fig. 1). This phenomenon is observed in simulations^15,19^ and experimental flow cells^18,20^ as well as in in vivo samples^18^. In this study, we use individual-based simulations of growing biofilms to investigate in detail the connection between the active layer and the biofilm-fingering transition. We develop a computational method that allows us to simulate biofilm growth over long times, to obtain a clear picture of the steady-state spatial structure. We observe three qualitatively different types of biofilm growth, each with a distinct active layer behaviour and interface roughness trajectory. These growth types are distinguished qualitatively by their active layer dynamics. We show that the formation of gaps in the active layer can lead to local parts of the interface ‘pinning’, or becoming stationary and falling behind the advancing biofilm interface. These pinning sites, which can be transient or permanent, ultimately lead to the fingering of the interface. Therefore we argue that, while the average active layer thickness is important, active layer dynamics also play a key role in the fingering transition. Framing our results in the form of a phase diagram, we find that the biofilm pinning transition is controlled by the relative fluctuations in the active layer thickness, i.e. by the ratio between the standard deviation of the active layer thickness and its average. Interestingly, this corresponds closely with the combined parameter proposed by ref. ^11^. Since the standard deviation reflects fluctuations in the active layer thickness, this supports the hypothesis that active layer dynamics play a key role in driving the spatial structure of the growing biofilm interface.

## Results

### Individual-based simulations produce distinct biofilm morphologies

To investigate biofilm morphology, we performed individual-based simulations of biofilm growth using the well-established iDynoMiCS simulation software^21^. Our simulations model individual bacteria as discs (in 2D) which consume nutrients, grow, divide, and push each other apart (see Methods). The nutrient is assumed to diffuse from above, mimicking approximately an experimental flow-cell setup (see Methods)^21^. We performed a grid of simulations, varying systematically the bulk nutrient concentration *S*_bulk_ and the maximum specific growth rate of the bacteria *μ*_max_. These parameters could, in principle, be controlled experimentally by changing the nutrient concentration of the fluid medium in a flow cell setup, and the bacterial strain. All other parameters remain fixed in our simulations (see Table 1).

**Table 1:** Table of the input values used in our iDynoMiCS biofilm simulations.

Parameter	Values	Description	References
*S*_*bulk*_	6.6 × 10^−3^ g/liter	Bulk concentration of limiting growth resource (oxygen)	Saturation concentration of water at 37 ^o^C^56^
*Y*	0.64 g ⋅ g^−1^	Yield - grams of biomass produced per gram of oxygen consumed	^57^
*μ*_*max*_	0.29 1/h	Maximum specific growth rate	^57^
*k*_*S*_	8.12 × 10^−4^ g/liter	Concentration of oxygen at which the growth is half maximal	^2^
*D*_*S*_	2.3 × 10^−4^ m^2^/day	Solute (oxygen) diffusion coefficient	^58^
Biofilm Diffusivity	0.8	Factor multiplying *D*_*S*_ to give solute diffusion coefficient inside the biofilm	^58,59^
*h*	80 μm	Diffusion boundary layer height	^13,60,61^
*ρ*_*B*_	200 g/liter	Biomass density of bacteria	^60,62^
*r*_div_	2 μm	Average cell maximum (division) radius	^57^
*k*_Shov_	1.15	‘Shove factor’ which multiplies the cell’s radius to give the radius of the ‘zone of influence’ for mechanical shoving	^21^
*L*_*y*_	1032 μm	Simulation width	
*N*_0_	300	Number of initialised cells	

These values aim to be consistent with *Pseudomonas aeruginosa* in an oxygen-limited flow cell type setup^2,51^.

The overall biofilm growth rate (cell number vs time) depends strongly on our simulation parameters, with small values of *S*_bulk_, or large values of *μ*_max_, leading to slow growth (Supplementary Fig. 1). Therefore, to make a fair comparison between biofilms at a similar developmental stage, we chose to compare simulated biofilms of the same size (cell number), rather than age (time)^22^.

**Fig. 2:**
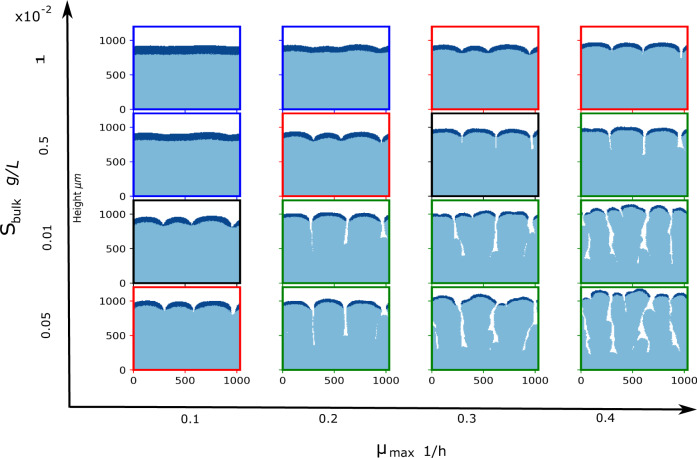
Emergence of distinct biofilm morphologies. Snapshots from our grid of simulations, for biofilm sizes of ~75,000 cells. Our grid of simulations was defined by varying the bulk nutrient concentration *S*_bulk_ and maximum specific cell growth rate *μ*_max_. The remainder of the simulation input parameters are held constant, and detailed in Table 1. In the snapshots, the active layer is shown in dark blue, while the inactive part of the biofilm is shown in light blue. The active layer is defined as described in the Methods section. The coloured borders around the snapshots indicate the phase of biofilm growth—blue for unpinned, red for transiently pinned, green for pinned and black for transitional, as defined in the text and in Figs. 4 and 5.

Figure 2 shows snapshots from our grid of simulations, for biofilms of 75,000 cells. A variety of different biofilm morphologies emerge, from approximately flat (high *S*_bulk_ and low *μ*_max_) to fingered (low *S*_bulk_ and high *μ*_max_). We identify cells as belonging to the active layer if their growth rate is greater than 1/1000 of the maximal growth rate possible in a given simulation *μ*_max_*S*_bulk_/(*k*_*S*_ + *S*_bulk_) (see Methods). In our simulations, we can compute the average thickness of the active layer, across the biofilm width (see Methods). After an initial period corresponding to biofilm formation, the average active layer thickness reaches a steady state value (Supplementary Figs. 2, 3), consistent with previous work^15–17,19^. As expected, the steady state value of the average active layer thickness varies for different simulation parameters (Fig. 2 and Supplementary Figs. 2–4). The active layer is, on average, thicker for the smooth biofilms, which correspond to high values of *S*_bulk_ and low values of *μ*_max_ and thinner for the fingered biofilms, which correspond to low values of *S*_bulk_ and high values of *μ*_max_^11,13,15,19^.

In our simulations, the active layer thickness can vary greatly between different local positions along the biofilm interface (Fig. 2). We quantify the extent of local variation in the active layer by computing the standard deviation of the active layer thickness across the biofilm width (see Methods for details). The standard deviation of the active layer thickness takes longer to reach its steady state than the average active layer thickness (Supplementary Figs. 2, 5), highlighting the need for long-time simulations such as those performed here. The standard deviation of the active layer thickness is larger in fingered biofilms (low *S*_bulk_ and high *μ*_max_) than in smooth biofilms (low *S*_bulk_ and high *μ*_max_). This reflects the fact that the active layer is unbroken in the smooth biofilms, but broken in the fingered biofilms, where growth only occurs at the tips of the fingers (hence the troughs correspond to gaps in the active layer).

### Dimensionless control parameter governs relative active layer fluctuations

**Fig. 3:**
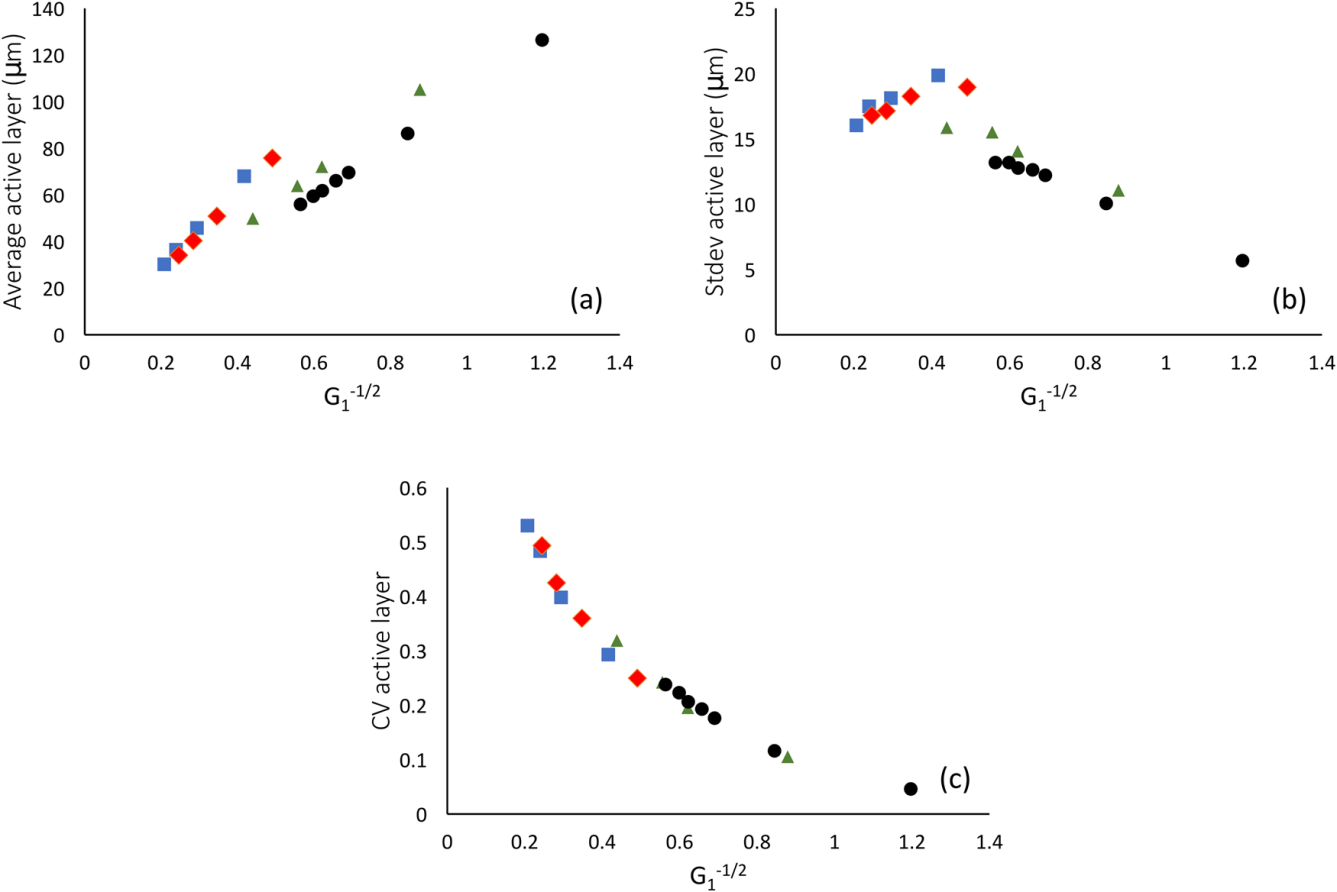
Dimensionless control parameter and the active layer. **a** Average active layer thickness (averaged across the biofilm width for steady-state simulations, see Methods), **b** Standard deviation of the active layer thickness, and **c** Coefficient of variation of the active layer thickness, i.e. standard deviation divided by the mean. In all panels, the parameter on the horizontal axis is the dimensionless combined parameter proposed by ref. ^11^, $\scriptstyle{G}_{1}^{-\frac{1}{2}}={({D}_{B}Y({k}_{S}+{S}_{{\mathrm{bulk}}})/({L}_{y}^{2}\rho {\mu }_{{\mathrm{max}}}))}^{\frac{1}{2}}$. The data points correspond to 19 simulations with different values of the parameters *S*_bulk_ and *μ*_max_. Data points are grouped according to their *S*_bulk_ values. Blue squares: *S*_bulk_ = 0.0005 g/L, *μ*_max_ = (0.1, 0.2, 0.3, 0.4)/h; Red diamonds: *S*_bulk_ = 0.001 g/L, *μ*_max_ = (0.1, 0.2, 0.3, 0.4)/h; green triangles: *S*_bulk_ = 0.005 g/L, *μ*_max_ = (0.1, 0.2, 0.25, 0.4)/h; black circles: *S*_bulk_ = 0.01 g/L, *μ*_max_ = (0.1, 0.2, 0.3, 0.33, 0.37, 0.4, 0.45)/h. See Supplementary Fig. 6 for an equivalent analysis with the alternative combined parameter $\scriptstyle{G}_{2}^{-\frac{1}{2}}$.

Previous studies^11,13,15^ have proposed that biofilm structure is controlled by the thickness of the active layer, which in turn is determined by the balance between nutrient transport and microbial growth. This balance can be described by a combination of model parameters; in particular, Dockery and Klapper^11^ suggested the dimensionless parameter $\scriptstyle{G}_{1}^{-\frac{1}{2}}={({D}_{B}Y({k}_{S}+{S}_{{\mathrm{bulk}}})/({L}_{y}^{2}\rho {\mu }_{{\mathrm{max}}}))}^{\frac{1}{2}}$. To test whether this parameter indeed controls the behaviour of the active layer in our simulations, we plotted the average active layer thickness (in steady state), its standard deviation, and its coefficient of variation (standard deviation divided by mean), versus $\scriptstyle{G}_{1}^{-\frac{1}{2}}$ (Fig. 3). Both the average and standard deviation of the active layer thickness are well predicted by the value of $\scriptstyle{G}_{1}^{-\frac{1}{2}}$, but the prediction is not perfect (Fig. 3a and b, respectively). However, the coefficient of variation is much better predicted by $\scriptstyle{G}_{1}^{-\frac{1}{2}}$ (Fig. 3c). Therefore, in our simulations, the combined parameter proposed by Dockery and Klapper actually describes the magnitude of the fluctuations in the active layer thickness, relative to the mean active layer thickness.

Other studies^13,15^ proposed other control parameters, in which *S*_bulk_ takes the place of the factor *k*_*S*_ + *S*_bulk_ (among other differences). We encapsulate this by defining another dimensionless parameter $\scriptstyle{{G}_{2}^{-\frac{1}{2}} = ({D}_{B}Y{S}_{{\mathrm{bulk}}})/({L}_{y}^{2}\rho {\mu }_{{\mathrm{max}}})\left.\right)}^{\frac{1}{2}}$. An equivalent analysis for the parameter $\scriptstyle{G}_{2}^{-\frac{1}{2}}$ produces similar conclusions, but the correlation is somewhat less good than for $\scriptstyle{G}_{1}^{-\frac{1}{2}}$ (Supplementary Fig. 6).

### The biofilm structural transition can be defined by interface pinning

**Fig. 4:**
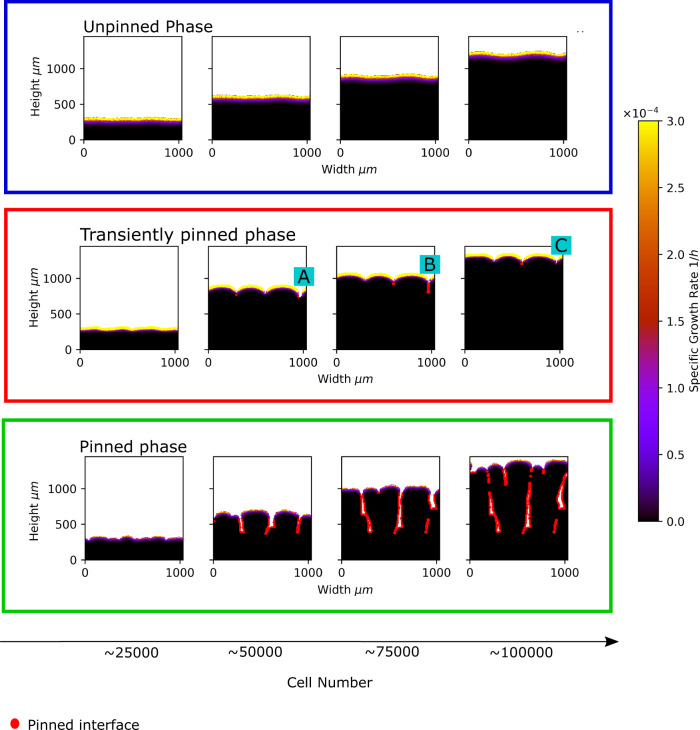
Distinct phases of biofilm dynamics. Biofilm growth is illustrated for three parameter sets, representing three qualitatively different types of biofilm behaviour, or `phases'. The top row of snapshots is for parameters *S*_bulk_ = 0.01 g/L, *μ*_max_ = 0.1 1/h, which represent the unpinned phase. The central row of snapshots is for parameters *S*_bulk_ = 0.01 g/L, *μ*_max_ = 0.4 1/h, representing the transiently pinned phase. The bottom row of snapshots is for parameters *S*_bulk_ = 0.0005 g/L, *μ*_max_ = 0.4 1/h and represents the pinned phase. Biofilm growth is shown from left to right; snapshots are shown for biofilm sizes of 25,000, 50,000, 75,000 and 100,0000 cells. In the snapshots, cells are colour-coded according to their specific growth rate. Parts of the interface that are pinned (i.e. have not moved in the previous six hours of simulated time) are represented in red. The parts of the interface labelled A, B and C in the figure correspond respectively to a gap in the active layer, a pinning site and a gap in the active layer gap after a pinning site has closed.

**Fig. 5:**
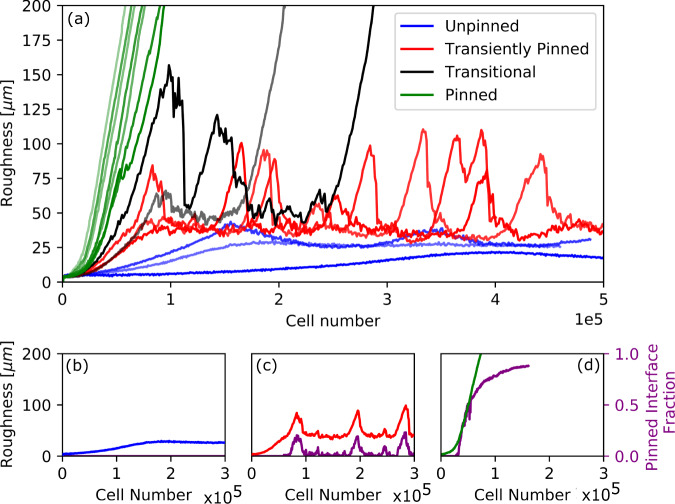
Biofilm roughness dynamics driven by interface pinning. Panel **a** shows roughness trajectories (roughness vs cell number) for each of the simulations of Fig. 2, i.e. for varying *S*_bulk_ and *μ*_max_. Trajectories in blue, red and green correspond to simulations in the unpinned, transiently pinned and pinned phases, respectively, as defined by their interface pinning behaviour (see Fig. 4). The trajectories shown in black and grey appear to be transitioning between phases. Panels **b**–**d** show a sample roughness trajectory for each of the phases (blue/red/green line) plotted together with the corresponding trajectory for a fraction of the interface that is pinned, i.e. that has not moved in the previous 6 hours of simulated time (purple line). Panels **b**–**d** show the same simulations for each phase as Fig. 4, i.e. *S*_bulk_ = 0.01 g/L, *μ*_max_ = 0.1 1/h for the unpinned phase, *S*_bulk_ = 0.01 g/L, *μ*_*max*_ = 0.4 1/h for the transiently pinned phase and *S*_bulk_ = 0.0005 g/L, *μ*_max_ = 0.4 1/h for the pinned phase. Note that for the unpinned phase (**b**) the blue and purple curves are indistinguishable. Supplementary Figs. 7 and 8 show the same trajectories of interface roughness and pinned interface fraction for all of our simulations individually.

We observe three distinct types of biofilm structures, which are also associated with different interfacial dynamics. For large *S*_bulk_ and small *μ*_max_ the biofilm interface is smooth (Fig. 4, top). Quantifying the interface roughness as the biofilm grows (accounting for overhangs; see Methods) shows that the roughness quickly reaches a steady state at a low value (Fig. 5a, blue trajectories). In these simulations the active layer is unbroken (Fig. 4, top). For intermediate *S*_bulk_ and small *μ*_max_ the interface is less smooth, and the active layer shows transient gaps, i.e., regions along the interface where there are no growing cells (Fig. 4, middle, point A). The interface roughness reaches a steady state as the simulation progresses but the steady state is characterised by dramatic fluctuations (Fig. 5a, red trajectories). Finally, for small *S*_bulk_ and large *μ*_max_, the interface becomes fingered. These simulations show permanent gaps in the active layer (Fig. 4, bottom) and the interface roughness does not reach a steady state but instead increases monotonically throughout the simulation (Fig. 5a, green trajectories). We also observe some simulations where the interface roughness appears to transition between different dynamical behaviours (Fig. 5a, black and grey trajectories).

In our simulations, biofilm structure is linked with active layer dynamics via interface pinning. Pinning, in which part of a moving interface stops advancing and falls behind the rest of the moving front, is a well-known phenomenon in interface growth theory in statistical physics (see, e.g. ref. ^23^). In our simulations, a gap in the active layer produces a local region of the interface that is not growing, and so remains stationary as other parts of the biofilm grow (e.g. Fig. 4, middle, point B). This part of the interface is therefore pinned. Pinning directly impacts biofilm structure, since it creates a trough in the interface at the pinned region. This correlates with an increase in interface roughness (defined as the standard deviation of the interface height) since the surrounding parts of the interface continue to advance while the pinned region does not. Quantifying the fraction of the interface that is pinned (i.e. stationary for at least 6 hours of simulated time; see Methods for details), we find a close correlation between trajectories of the interface roughness and the pinned interface fraction (Fig. 5b–d; see also Supplementary Figs. 7, 8). Our simulations may show no pinning (if the active layer remains unbroken), correlating with a smooth interface (Fig. 5b), or they may show transient pinning (where active layer gaps form and close up again, e.g. Fig. 4, middle, point C), corresponding to strong fluctuations in the interface roughness (Fig. 5c), or they may form permanently pinned regions of the interface, leading to biofilm-fingering and monotonically increasing roughness (Fig. 5d). Interestingly, the interface roughness is correlated with the standard deviation of the active layer thickness when the interface is unpinned, but not when it is pinned; see Supplementary Fig. 9.

The central role of interface pinning in structure formation motivates us to classify different simulations according to their pinning behaviour. Inspired by the concept of the pinning transition in interface growth theory in statistical physics^24^, we class our simulations as being in the ‘unpinned phase’ (unbroken active layer, no pinning), the ‘transiently pinned phase’ (transient active layer gaps, transient pinning), or the ‘pinned phase’ (permanent active layer gaps and pinning; fingered biofilm).

### Pinning happens when active layer gaps merge

**Fig. 6:**
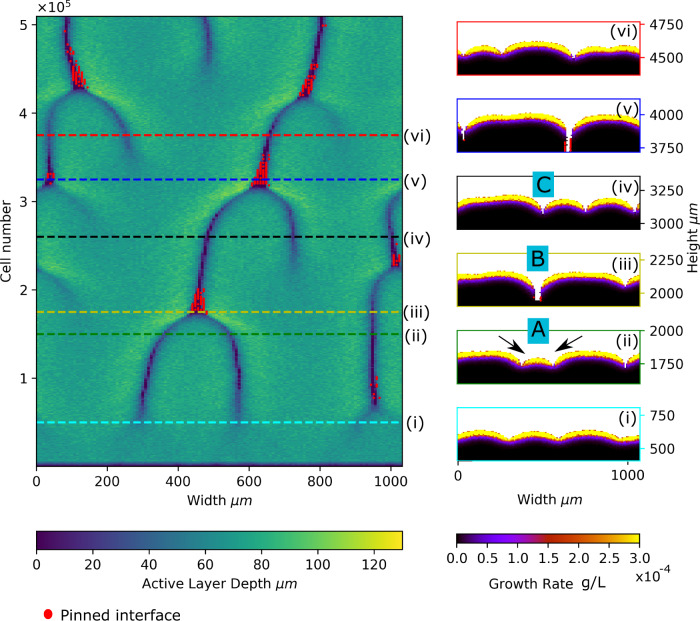
Active layer gap dynamics and pinning. The left panel shows an active layer kymograph plot for a simulation in the transiently pinned phase (*S*_bulk_ = 0.005 g/L, *μ*_max_ = 0.2 1/h). The local active layer thickness is plotted along the biofilm width (horizontal axis). Active layer thickness is indicated by the colour scale, such that darker colours represent regions with thin or no active layer and lighter colours represent regions with a thicker active layer. The vertical axis represents the number of bacterial cells in the biofilm (i.e. the total biomass), revealing the dynamics of the active layer as biofilm growth progresses. In the kymograph, gaps in the active layer are visible as dark lines. The red dots indicate local regions of the interface that are pinned (see Methods). Snapshots (i–vi) on the right-hand side correspond to the dashed lines on the kymograph. Labels A, B and C indicate regions of the interface corresponding to just before, during and after the formation of a pinning site. In particular, a small bulge in the interface (labelled A), bordered by two active layer gaps (arrows) is engulfed by the lateral expansion of surrounding, larger bulges (B and C).

Tracking the dynamics of the active layer along the biofilm interface provides a more detailed picture of the mechanism underlying interface pinning. An active layer kymograph plot for a simulation in the transiently pinned phase provides a convenient way to visualise the creation, annihilation, and motion of active layer gaps (Fig. 6). The kymograph reveals that active layer gaps appear spontaneously (emergence of a new dark line reading bottom to top in the kymograph), but they disappear only by merging with other active layer gaps. We also observe the motion of active layer gaps (sloping of the dark lines in the kymograph), due to pushing between adjacent bulges in the interface (since active layer gaps correspond to the troughs between bulges). This motion is clearly not diffusive (Fig. 6; see also Supplementary Fig. 10 for the kymographs of our complete grid of simulations).

Superposing the location of pinning sites onto the active layer kymograph (Fig. 6) allows us to connect the dynamics of the active layer to interface pinning. In this simulation, frequent formation and annihilation of pinning sites occurs. Strikingly, new pinning sites are formed when two active layer gaps merge. Inspection of the corresponding simulation snapshots shows that the merger of two active layer gaps occurs when a small bulge in the interface is engulfed by the lateral expansion of surrounding, larger, bulges (Fig. 6). In the simulation of Fig. 6, the ‘bulge engulfment’ event that leads to active layer gap merging and formation of a new pinning site is accompanied by the appearance of a new active layer gap at a different point along the interface. Supplementary Fig. 10 confirms that this behaviour is common in the transiently pinned phase.

In contrast, the annihilation of a pinning site happens when a trough in the interface closes up. Careful inspection of movies from our simulations (see Supplementary movies) shows that this occurs because of lateral expansion, i.e. the trough becomes narrower from the sides. This lateral expansion appears to be driven by mechanical pushing interactions that are transmitted from the growing cells at the biofilm front down to the non-growing cells in the walls of the trough.

### Towards a phase diagram for biofilm pinning

Our work suggests that a growing biofilm can undergo a transition from a smooth, ‘unpinned’ state to a rough ‘transiently pinned’ or ‘pinned’ state. To understand better the nature of this transition, we plot a phase diagram. A phase diagram is a central concept in the physical sciences, used to describe how a system transitions from one state to another. The phase diagram shows how the state of the system changes as the environment changes. The environment is described by a ‘control parameter’, while the state of the system is described by an ‘order parameter’^25,26^. For example, in the classic textbook case of the magnetisation transition of a magnetic material, the control parameter is the temperature of the material and the order parameter is its degree of magnetisation. The nature of the order parameter and control parameter, and the shape of the resulting phase diagram, can reveal important information about the key physical principles underlying the transition (Supplementary Figs. 11,12). Although phase diagrams are usually used to describe systems at thermodynamic equilibrium, they can also provide useful insights for non-equilibrium, driven systems; see, e.g. refs. ^17,27–30^.

To construct a phase diagram for interface pinning in our simulations, we therefore aim to plot an order parameter, describing the extent of interface pinning, as a function of a control parameter. As our order parameter we choose the average steady-state fraction of the interface that is pinned (see Methods). This is a well-defined quantity that takes different values in the three phases of biofilm growth. In the unpinned phase it is zero, while it lies in the ranges 0.010–0.284 and 0.741–0.875 for our simulations in the transiently pinned and pinned phases, respectively.

**Fig. 7:**
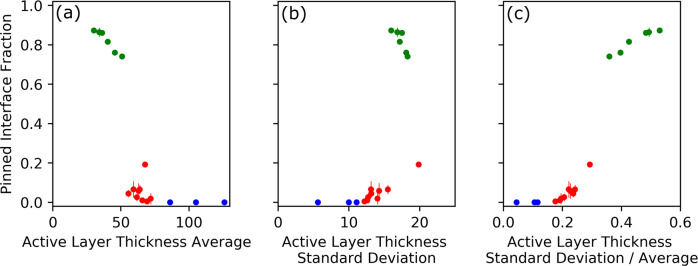
Phase diagram for the pinning transition. In each panel, our chosen order parameter, the average steady-state pinned interface fraction, is plotted against three potential control parameters for the data from our simulations. These are the average active layer thickness (**a**), the steady state of the standard deviation of the active layer thickness (**b**), and the steady state coefficient of variation (i.e. standard deviation/mean) of the active layer thickness (**c**). Simulation data points are coloured according to whether the simulation is in the unpinned (blue), transiently pinned (red) or pinned (blue) phase. The simulations included in our plot are as in Fig. 4, but the transitional simulations (see Fig. 5) are not included. The simulation with *μ*_max_ = 0.4 1/h, *S*_bulk_ = 0.005 is also not included, as it had not fully reached the steady state (see Supplementary Fig. 8). To gain resolution in the transition region, we also included additional simulations in the transiently pinned phase, with parameter values of *μ*_max_ = 0.3 1/h, *S*_bulk_ = 0.007 g/L; *μ*_max_ = 0.33 1/h, *S*_bulk_ = 0.01 g/L, *μ*_max_ = 0.45 1/h, *S*_bulk_ = 0.01 g/L; *μ*_max_ = 0.25 1/h, *S*_bulk_ = 0.005 g/L; *μ*_max_ = 0.37 1/h, *S*_bulk_ = 0.01 g/L. Error bars are calculated using the standard error of the mean for correlated data (see Methods).

Our observations so far suggest that interface pinning is closely coupled to the dynamical behaviour of the active layer (e.g. Fig. 6). Therefore, as candidate control parameters in our phase diagram, we tested the steady-state values of the average active layer thickness, the standard deviation of the active layer thickness, and the coefficient of variation of the active layer thickness (Fig. 7a–c) respectively). We define a control parameter as optimal if it causes our simulation data to collapse onto a single curve in the control parameter - order parameter space.

As a candidate control parameter, the average active layer thickness does distinguish between the three phases, but it does not produce a perfect collapse of the data onto a single line, particularly in the transiently pinned phase (Fig. 7a). This suggests that the average active layer thickness is not the only factor controlling the pinning transition.

The standard deviation of the active layer thickness provides a crude measure of active layer fluctuations. As a candidate control parameter, it also distinguishes between the three phases, but the transiently pinned phase data does not collapse perfectly, and the pinned phase data shows a strange behaviour where the order parameter decreases with increasing control parameters (Fig. 7b).

Finally, we tested the coefficient of variation of the active layer thickness, i.e. the standard deviation divided by the mean, as a control parameter. This makes intuitive sense, since we expect that the creation of an active layer gap (which can lead to a pinning site) requires the active layer thickness to fluctuate by an amount that is comparable to its mean value. We are also motivated by the fact that this quantity correlates well with the dimensionless combined parameter of Dockery and Klapper^11^ (Fig. 3c). This candidate control parameter produces a much better data collapse than our previous candidate control parameters (Fig. 7c). As the coefficient of variation of the active layer thickness increases, the system transitions from the unpinned to the transiently pinned to the pinned phase. Therefore we conclude that (i) active layer fluctuations are important in driving the biofilm pinning transition and (ii) the magnitude of these fluctuations relative to the mean active layer thickness is the relevant quantity.

## Discussion

In this work, we used individual-based computer simulations to investigate the spatial structure of growing bacterial biofilms. Our simulations include the effects of local nutrient limitation (modelled via a reaction-diffusion equation) and mechanical pushing between the cells (modelled via a ‘shoving’ algorithm; see Methods). Varying the nutrient concentration and the maximal specific growth rate of the bacteria, we observed a diversity of biofilm morphologies, ranging from smooth to highly-fingered interfaces. The active layer of growing cells at the biofilm interface plays a central role in biofilm morphology; previous work has suggested that the balance between nutrient transport and consumption controls active layer thickness; this balance can be expressed by a dimensionless combined parameter^11,13,15^. Interestingly, in our simulations, the dimensionless parameter correlated better with the relative fluctuations of the active layer thickness than with the mean active layer thickness. This suggests that active layer dynamics play an important role in driving biofilm structure; a conclusion that is supported by a detailed analysis of our simulations. Collisions between local gaps in the active layer lead to interface pinning, in which a part of the interface stops growing relative to the rest of the biofilm. Interface pinning then leads to fingering.

Our simulations could be classified into three ‘phases’ of biofilm growth. In the ‘unpinned’ phase, the interface is smooth and does not pin, and the active layer is thick and unbroken. The ‘transiently pinned’ phase is characterised by the appearance of transient local pinning sites along the interface and large temporal fluctuations in the interface roughness. In the ‘pinned’ phase, the interface develops fingers, which arise from pinning sites that appear but do not disappear; correspondingly, the interface roughness increases throughout the simulation. Using the coefficient of variation of the active layer thickness as a control parameter, we were able to plot a phase diagram for biofilm pinning. The finding that the coefficient of variation of the active layer thickness is a better control parameter than the mean strengthens our view that fluctuations in the active layer are important in controlling biofilm spatial structure.

The form of the phase diagram can also tell us about the underlying nature of a phase transition. Statistical physics distinguishes ‘discontinuous’ transitions, in which the order parameter jumps discontinuously from zero to a finite value at a critical value of the control parameter, from ‘continuous’ transitions, in which the order parameter changes continuously from zero to a finite value as the control parameter varies (Supplementary Fig. 11)^25^. This distinction has relevance for the kinetics of the phase transition since, for equilibrium systems, a discontinuous transition implies that stochastic fluctuation is required to overcome an activation barrier (i.e. it is a nucleated process), while for a continuous transition, the transition happens spontaneously^25^ (Supplementary Fig. 12). A similar picture can hold for non-equilibrium phase transitions; see, e.g. ref. ^31^. In practice, the distinction between discontinuous and continuous transitions can become blurred by finite size effects (one only observes a true discontinuity in the phase diagram for systems of infinite size)^32^.

In our phase diagram, there is a large jump in the value of the order parameter between the transiently pinned and pinned phases (Fig. 7c). Therefore, we tentatively suggest that this may be a discontinuous transition, with the apparent smoothing arising from finite size effects. In this discontinuous transition scenario, the transiently pinned phase would arise only in systems of finite size (including real biofilms), while a hypothetical biofilm of infinite lateral size would transition directly from the unpinned to the pinned state upon varying the control parameter. This might also suggest that the transition to a pinned state is a nucleation phenomenon (Supplementary Fig. 12), such that a critical fluctuation, e.g. the appearance of a gap in the active layer that is wide enough that it does not close up again, may be needed to initiate biofilm fingering (Supplementary Fig. 12). This point could be clarified in future simulations by systematically varying the system size.

Our study is limited to parameter sets for which the active layer is thickness is at least several cell diameters. For extreme parameter sets (very small *S*_bulk_ or very large *μ*_max_), a different type of biofilm morphology can emerge, in which the fingers split into multiple branches. This phenomenon shows apparent similarity with diffusion-limited aggregation in statistical physics^33^, and may be worthy of investigation in future work.

In this work, we have taken care to study biofilm growth over long times, once a steady state has been reached; this required the development of a computationally efficient clipping algorithm (see Methods and Supplementary Material). We note that at earlier times in our simulations, the interface roughness can appear to reach a plateau (even in the pinned phase), before later increasing (Supplementary Fig. 13). Therefore, in shorter simulations, it may be hard to know whether the true steady state has been reached. Our long-time simulations suggest that, in the pinned phase, the interface roughness does not, in fact, reach a steady state but rather continues to increase because the tips of the fingers continue to grow while the interface remains pinned at the troughs. In contrast, a finite steady-state roughness would correspond to an interface that has stalled in its net growth. From a practical point of view, the fact that the biofilm fingers continue to grow in our simulations presents computational issues since the use of our clipping algorithm is constrained in the case of fingered biofilms (see Methods). This means that while the steady state of the active layer dynamics and interface roughness behaviour can be reached, it remains challenging to reach the full steady state of the pinned interface fraction in the case of the pinned biofilms (see, e.g. Supplementary Fig. 8).

Our simulations are performed in two dimensions, for reasons of computational feasibility. The dimensionality of a system can have profound effects on phase transitions^24^: therefore, it will be important to determine in future work whether the same phenomena occur in 3D models. We also note that the representation of mechanical interactions in our simulations is rather crude (the iDynoMiCS algorithm simply resolves overlaps due to growth by a random ‘shoving’ algorithm; see Methods and ref. ^21^). Other studies have represented mechanical interactions in more detail^14,34–36^; use of such algorithms may lead to deeper insight into the role of mechanical interactions in the pinning transition.

Our work has an interesting analogy with pattern formation in crystal growth, where complex crystal morphologies arise from instabilities in the advancing solidification front^37^. The local rate of crystal growth can be limited by the rate of diffusion of heat away from the crystallisation front (e.g. crystallisation of small molecules or metals), or by the rate of diffusion of molecules to the growth front (e.g. in polymer crystallisation). This leads to fingering instabilities similar to the nutrient-driven fingering instability in biofilm growth^11^. The emergence of different crystal forms might also have parallels with the emergence of mutant clones in a biofilm. However, we note that in crystal growth, the morphological instability is ultimately limited by surface tension, which tends to smooth the interface^37^, while the limiting factor for biofilm growth is less clear (although it probably involves mechanical interactions). We also note that in a crystal, growth occurs only right at the interface, while for a biofilm, there is a growing region of finite thickness at the interface (the active layer).

Our work also has a clear connection with the statistical physics of pinning-depinning transitions in interface growth. Here, diverse interface growth phenomena are grouped into a small number of ‘universality classes’, based on their scaling behaviour (the values of the exponents in plots of, e.g. roughness vs time). Phenomenological stochastic differential equations are then used to describe interface growth within a particular universality class; for example, the classical Kardar–Parisi–Zhang (KPZ) equation describes fluctuations of an unpinned growing interface, while the addition of a quenched noise term to the KPZ equation leads to a model for interface pinning. This ‘quenched KPZ (qKPZ)’ model shows a flat phase with no pinning sites, a pinned phase and an intermediate phase in which pinning sites are overcome^24,38^ (although it is unclear whether this model predicts monotonically increasing roughness in the pinned phase). The qKPZ equation has been applied to biofilm growth^39,40^, but the source of the quenched noise is usually ascribed to external inhomogeneities in the environment. In our simulations, there are no such inhomogeneities; rather, pinning arises from the spontaneous emergence of gaps in the active layer. In the interface growth theory literature, KPZ-type models also exist where interface pinning arises from internal fluctuations in the growth process^38,41,42^, or where the growing interface is coupled to a non-equilibrium field (such as a nutrient field)^43^. However, the relevance of such models for bacterial biofilms and colonies has not been investigated. It is also possible that biofilm growth might be described by an alternative type of interface growth model, such as diffusion-limited aggregation^33^. While our focus here was on a more mechanistic analysis of the role of the active layer, it would certainly be interesting in future work to clarify the connection with interface growth theory by measuring the scaling exponents for biofilm growth in individual-based simulations.

From a biological point of view, our simulations are, of course, highly simplified. Perhaps most importantly, our model does not include the extracellular matrix (EPS), which means that our simulated biofilms have a much higher cell density than flow cell biofilms formed by, e.g. *Pseudomonas aeruginosa*. We would also expect the mechanical properties of the biofilm to be strongly influenced by EPS^44^. This could affect the predictions of the model, since, for example, EPS-mediated interactions might act non-locally (between cells relatively far apart in the biofilm), which could alter the phase behaviour. Attachment and detachment of planktonic bacteria from the biofilm^6^, and possible external forces acting on the biofilm (e.g. from host tissue or mucus as in infected cystic fibrosis lung tissue^45^) are also expected to strongly affect the spatial structure. We also neglect many other features of real biofilms, such as fluid flow, chemical signalling between cells and phenotypic changes associated with biofilm growth. The size of our simulated biofilms is also unrealistic. In order to reach the steady state, which is necessary for a rigorous analysis of the underlying physics, our simulations generate extremely thick biofilms, much thicker than those seen in experimental flow cell experiments.

Routine characterisation of surface roughness in confocal laser-scanning microscopy images of flow-cell biofilms is now possible^46,47^. Previous experimental studies of biofilm development have mainly focused on early-stage biofilms, where mechanisms such as collective surface motion^48^, transitions between 1, 2 and 3-dimensional forms^22,49,50^ and biofilm seeding from preformed aggregates^2,51^ have been discussed. Motility can also play a role in later-stage biofilms, where mushroom-shaped structures can form in which non-motile bacteria form ‘stalks’ while motile bacteria form ‘caps’^3^. Up to now, few studies of dynamical tracking of changes in biofilm structure, such as the formation and annihilation of bulges in the growing interface that we see in the ‘transiently pinned phase’, have been performed for mature biofilms. Our study suggests that such analysis, while technically challenging, could lead to interesting insights—although it is clear that biological mechanisms, including cell motility, that are not considered in our study, may prove to be important.

Despite the simplicity of the model that has been studied in this work, our simulations reveal fundamental insights into the spatial structure of growing biofilms. Specifically, our work points to pinning of the growing interface, as a driver for spatial structure. Furthermore, our simulations reveal a key role for the dynamics of the active layer in driving the creation and annihilation of pinning sites at the biofilm interface, resulting in transitions in spatial structure, with drastic effects on the interface roughness.

## Methods

### Simulation methods

In this work, we use individual-based biofilm modelling software iDynoMiCS^21^. Briefly, iDynoMiCS models the bacteria in a biofilm as individual agents whose behaviour is coupled to a solute reaction-diffusion equation^21^. The agents, which are assumed to be discs in continuum 2D space, each grows with a specific growth rate *μ* according to the Monod equation:

1 $$\mu ={\mu }_{{\mathrm{max}}}\frac{S}{{k}_{S}+S},$$

where *μ*_max_ is the maximum specific growth rate of the bacteria, *k*_*S*_ is the concentration of the solute at which the growth is half maximal, and *S* is the local solute concentration of the bacterial cell^52^. Once a bacterial cell reaches a maximum radius (which has a stochastic element), it divides into two daughters. Bacteria interact with one another mechanically via a shoving algorithm. Briefly, this algorithm detects pairs of bacteria whose ‘zones of influence’ (defined to be the radius multiplied by a ‘shove parameter’) overlap, and shuffles bacterial positions to avoid such overlaps^21^. Although iDynoMiCS has the facility to model extracellular matrix (EPS) as non-replicating particles, we did not model EPS in this study. In iDynoMiCS, the solute is represented by a concentration field which varies in space and time due to diffusion and consumption by the bacteria. A separation of timescales is assumed, such that the reaction-diffusion equation for the solute is assumed to reach a steady state faster than the timescale for bacterial growth; hence the solute concentration equations are solved to a steady state for each interaction of the bacterial growth updates. The computational domain is set up to resemble a flow cell, where the biofilm grows on a hard surface and nutrients diffuse from above. Convective flow is not modelled, but rather it is assumed that there is a stationary layer of fluid close to the biofilm (the ‘boundary layer’)^21,53^. It is also assumed that the diffusion constant for solute is reduced inside the biofilm by a fixed factor compared to outside the biofilm. The input values we use in our simulations are based on experimental values for oxygen-limited *Pseudomonas aeruginosa* biofilms, as outlined in Table 1. The iDynoMiCS start files used in this study are publicly available (see section ‘Code availability’).

In order to reach long simulation timescales, we also use an additional ‘clipping’ algorithm in combination with iDynoMiCS. This algorithm periodically removes inactive cells far below the growing front, such that a computationally feasible number of cells remain in the simulation space. This is achieved by pausing the iDynoMiCS simulation and removing the relevant cells, or ‘clipping’, and then restarting the simulation. This clipping procedure is done at regular time intervals, such that each complete biofilm simulation consists of *N* segments, each of length (in time) *T*_*s*_, producing a total simulated time *T* = *NT*_*s*_. In the clipping procedure, bacterial cells, which are both below the lowest actively growing cell and below the minimum point of the interface (which can be different points depending on the biofilm configuration), are removed. The complete algorithm is shown in the Supplementary Information and the clipping code is publicly available (see section ‘Code availability’). We rigorously tested this algorithm to ensure it does not perturb biofilm growth (Supplementary Figs. 14, 15).

### Characterisation of spatial structure

As we have seen, our analysis focuses on the active layer, which we define as the layer of growing cells at the top of the biofilm. More specifically, we begin by defining a threshold growth rate; cells which grow faster than this rate are defined to be part of the active layer. We consider a cell to be in the active layer when it grows at greater than 0.1% of the maximal specific growth rate *μ* = *μ*_max_*S*_bulk_/(*k*_*S*_ + *S*_bulk_) that is possible under the conditions of the simulation (i.e. for given values of *μ*_max_ and *S*_bulk_). Therefore the condition for a cell to be part of the active layer is

2 $$\mu\, > \,\frac{{\mu }_{{\mathrm{max}}}}{1000}\frac{{S}_{{\mathrm{bulk}}}}{{k}_{s}+{S}_{{\mathrm{bulk}}}}.$$

We now outline how the average and standard deviation of the active layer thickness are calculated. We define a grid spanning the simulation domain with *D* columns (horizontal bins) and *H* rows (vertical bins) of width 8 μm. For each of the *D* columns, we find the total number of ‘active’ grid squares whose biomass has an average specific growth rate above the threshold in Equation (2). This defines the local active layer thickness. For some biofilm configurations, for example, if the biofilm is rough, there can be a growing layer both at the leading edge of the biofilm, and within a trough, so the active grid squares are not necessarily adjacent to one another (Supplementary Fig. 16). Once the active layer width for each vertical strip has been found, the mean active layer thickness is found by averaging over all the *D* columns. The standard deviation of the active layer thickness is, in turn, calculated as the standard deviation of the active layer thickness of each of the columns i.e. it is the standard deviation of the local thickness of the active layer for a particular configuration. An active layer gap occurs when a vertical column on the grid contains no active grid squares.

Roughness is defined as the standard deviation of the height of the biofilm interface. In our simulations, especially in the pinned phase, we often see interface overhangs, in which the boundary of the biofilm has multiple values for a given position along the horizontal direction (see, e.g. the bottom right panels of Fig. 2). To correctly account for overhangs, we use a multi-valued interface^23,54^. On our grid of *D* 8 μm columns and *H* 8 μm rows, we search for grid squares which contain biomass but have a nearest neighbour that does not contain biomass. This produces a set of grid squares corresponding to the interface {*k*}, *k* = 1, ... *N*_int_ where *N*_int_ ≥ *D*. Defining the interface in this way allows there to be multiple vertical points for each horizontal point along the boundary—hence its description as a multi-valued interface (Supplementary Fig. 16).

We then define the interface width, i.e. the interface roughness, as the root mean square height of the points on the interface:

3 $$W(t)={\left\langle {\left[h(t)-\langle h(t)\rangle \right]}^{2}\right\rangle }^{1/2}.$$

Here, 〈*h*〉 is defined as the mean value of *h*_*k*_, where *h*_*k*_ is the vertical coordinate of the *k*th point along the interface, namely,

4 $$\langle h\rangle =\frac{1}{{N}_{int}}\mathop{\sum }\limits_{k=1}^{{N}_{int}}{h}_{k}$$

with *N*_*int*_ being the number of points along the interface.

We also define the stationary or pinned interface fraction. Here, we define the interface boundary as above, then look for parts of the interface which are both inactive and have not moved in a six-hour period (this being the frequency of output files). Specifically, we compare the positions of those interface grid squares which are inactive (i.e. they do not meet the condition of Eq. (2), with the interface boundary of the configuration 6h earlier. If this interface point is common to both configurations, it is defined to be both inactive and pinned. The pinned interface fraction *f*_*P*_ is then defined as

5 $${f}_{P}=\frac{{N}_{P}}{{N}_{{\mathrm{int}}}}$$

where *N*_*P*_ is the number of inactive, pinned interface points and *N*_int_ is the number of points on the interface.

The error bars in Fig. 7a–c are calculated as the standard error of the mean (SEM) of the *n* time points that were recorded for each variable (average active layer thickness, standard deviation of the active layer thickness and the pinned interface fraction) once the steady state had been reached, adjusted for the fact that our time series are correlated data. The auto-correlation time *τ* was calculated using code from ref. ^55^. The effective number of independent data points *n*_eff_ in our correlated data could be calculated as *n*_eff_ = *n*/*τ*. Finally, the standard error of the mean (SEM) was calculated as ${\mathrm{SEM}}=\sigma /\sqrt{{n}_{{\mathrm{eff}}}}$, where *σ* is the standard deviation of the *n* steady-state time points.

### Reporting summary

Further information on research design is available in the Nature Research Reporting Summary linked to this article.

## Supplementary information

Reporting Summary

Supplementary Material

Supplemental movies

## Data Availability

Datasets are also available from the corresponding author on reasonable request.

## Code availability

The clipping code and iDynoMiCS start files have been made publicly available via the Edinburgh DataShare repository and can be accessed using the following 10.7488/ds/3815.
