# Supplementary material for: Active layer dynamics drives a transition to biofilm fingering
Source: NPJ Biofilms Microbiomes. 2023 Apr 6;9:17. doi: 10.1038/s41522-023-00380-w (PMC10079924; doi:10.1038/s41522-023-00380-w)
Supplement: Supplementary file 2 — Supplementary Material [file 41522_2023_380_MOESM2_ESM.pdf]

Active layer dynamics drives a transition to biofilm fingering: Supplementary Information

Ellen Young, Gavin Melaugh and Rosalind J. Allen

Algorithm 1 The Clipping Algorithm. This procedure is used in combination with the iDynoMiCS software to reach long simulation times. This involves running many short simulations with the output biofilm configuration of one simulation segment being clipped and then used as the starting configuration of the next simulation segment. The conditions for determining the threshold height are based on the position of growing cells and on the position of the interface, to avoid perturbing the active layer or the biofilm interface.

1. Run the iDynoMiCS simulation from the starting configuration up to segment time T_s
2. Calculate the threshold height:
   (a) Calculate the minimum interface height and the minimum of the growing layer
   (b) Set the threshold height as the lowest of the minimum interface height and the minimum of the growing layer
   (c) Subtract a buffer of $20\mu m$ from the threshold height
   (d) If the threshold height is greater than 200 μm, set it to 200 μm.
3. Remove cells below the threshold height to create the clipped cell configuration
4. Restart the simulation using the clipped cell configuration, run up to time T_s
5. Repeat steps 2-5 N times until the end time $T = NT_s$ is reached

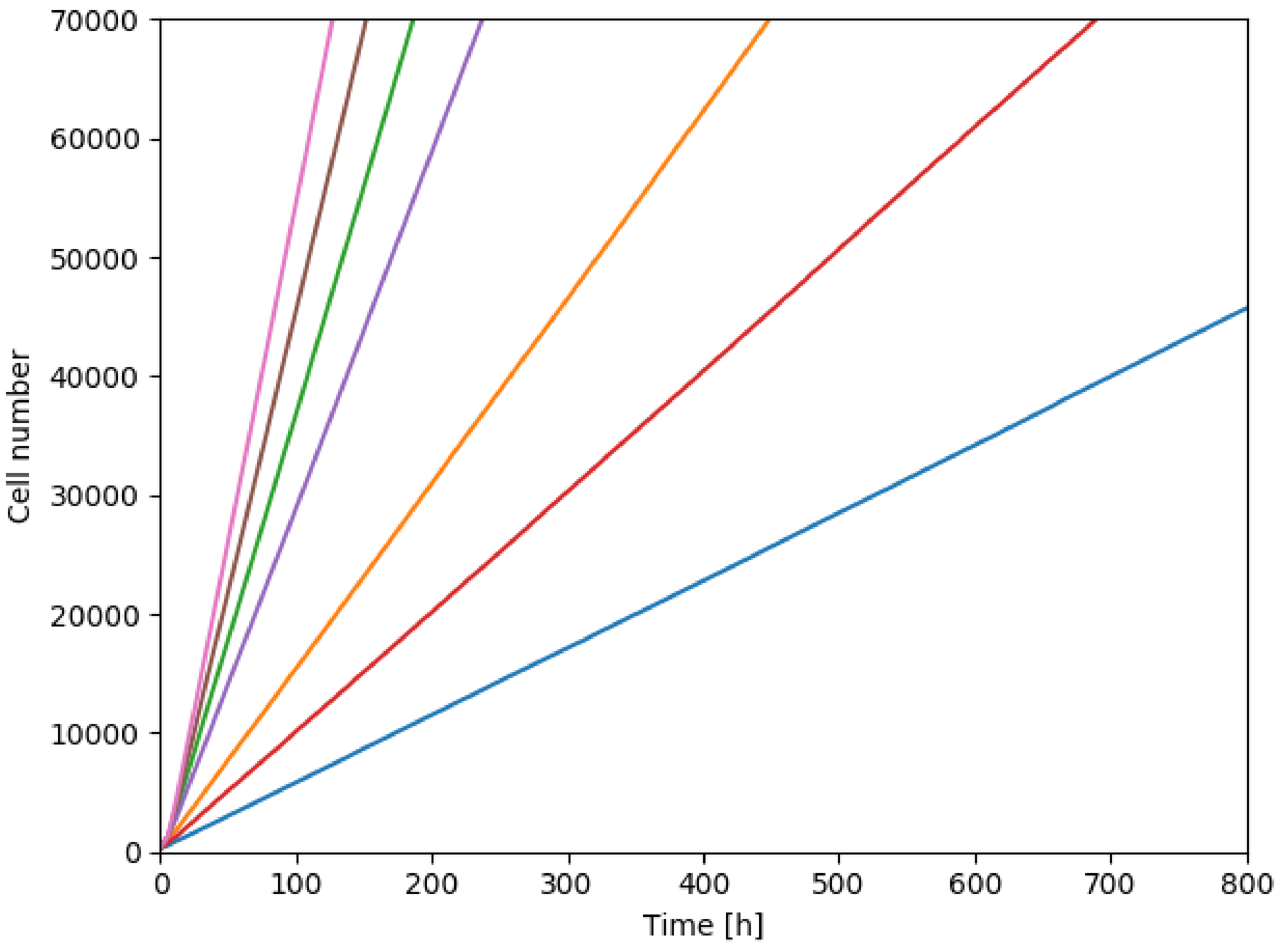

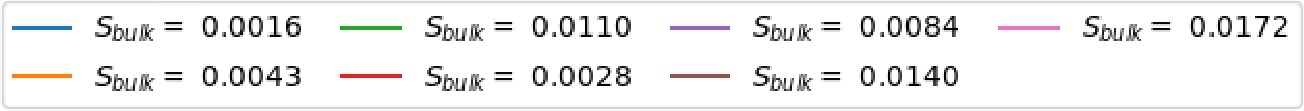

Supplementary Figure 1. Biofilm growth is linear in time Biofilm size, in terms of number of cells, is plotted as a function of time for simulations with varying bulk nutrient concentration S_{bulk}. The other input parameters are as specified in Table I of the main text. As detailed in the text, in the rest of our plots we use cell number as a proxy for time, with the growth rate (gradient of these plots) being the conversation factor between the two. In the legend, the units of S_{bulk} are g/L.

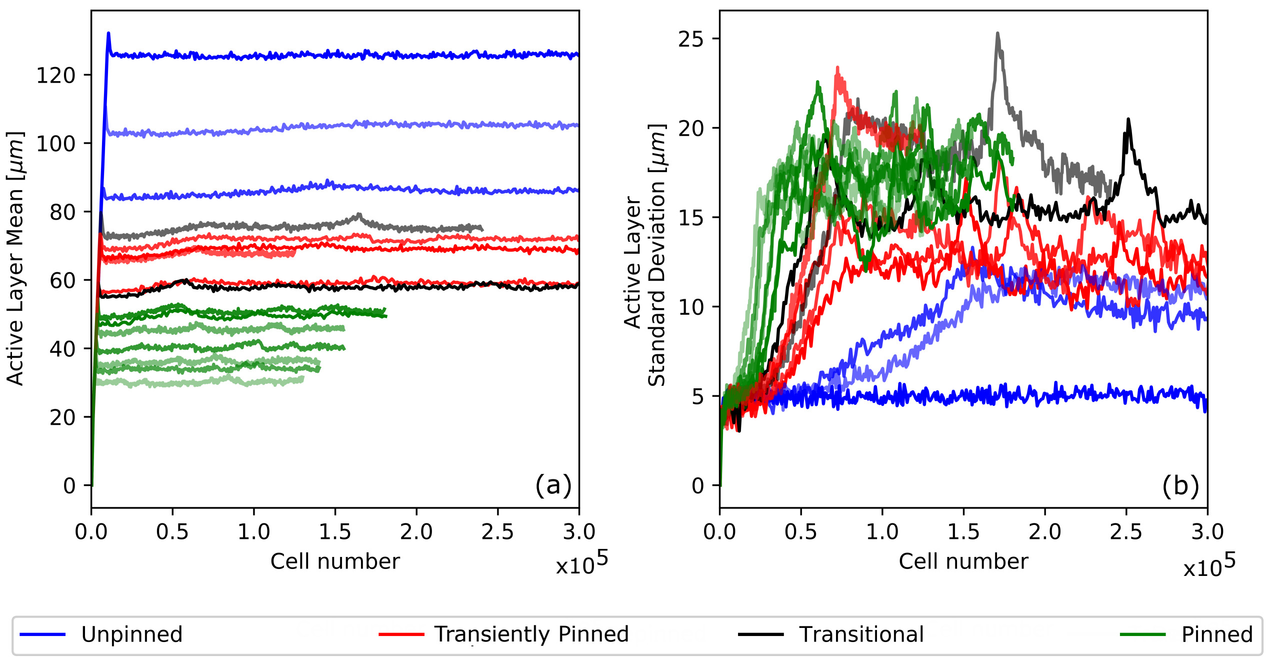

Supplementary Figure 2. Trajectories of the active layer thickness. The average (panel (a)) and standard deviation (panel (b)) of the active layer thickness are plotted as a function of cell number, for each of the simulations of Figure 4. Here cell number can be viewed as a proxy for time, with the conversion factor being the biofilm growth rate, which is parameter-dependent (see Supplementary Figure 1). The trajectories are colour coded according to their phase - blue for unpinned, red for transiently pinned, green for pinned and black/grey for transitional, as defined in the main text. Supplementary Figures 3 and 5 show these same trajectories individually.

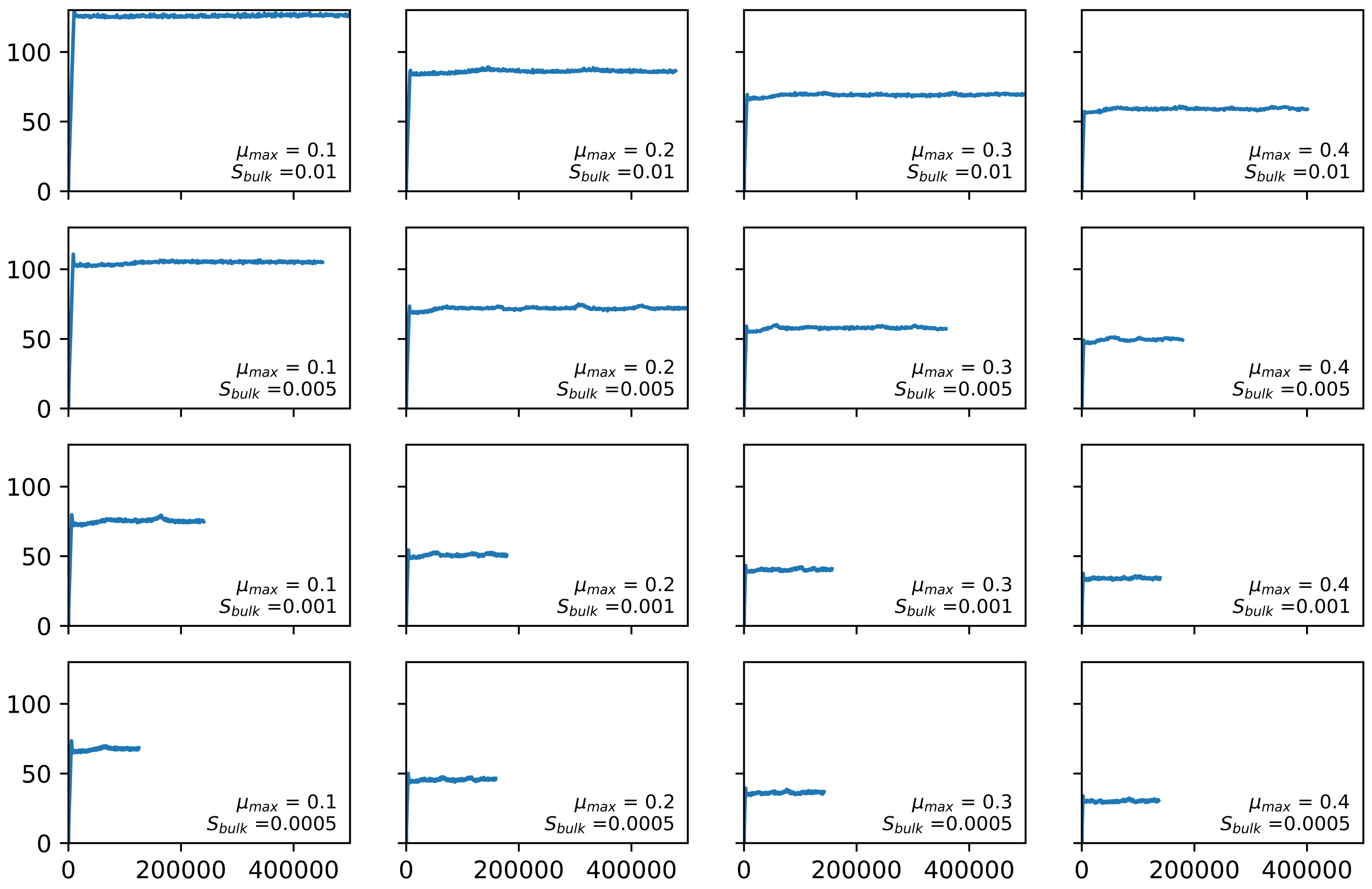

Supplementary Figure 3. Dynamics of the mean active layer thickness. The active layer thickness, averaged across the biofilm interface, is plotted as a function of biofilm size for all our simulations (i.e. for simulations with different values of μ_{max} and S_{bulk}; the other input parameters are as in Table I of the main text). In the legends, the units of μ_{max} are h^{-1} and units of S_{bulk} are g/L.

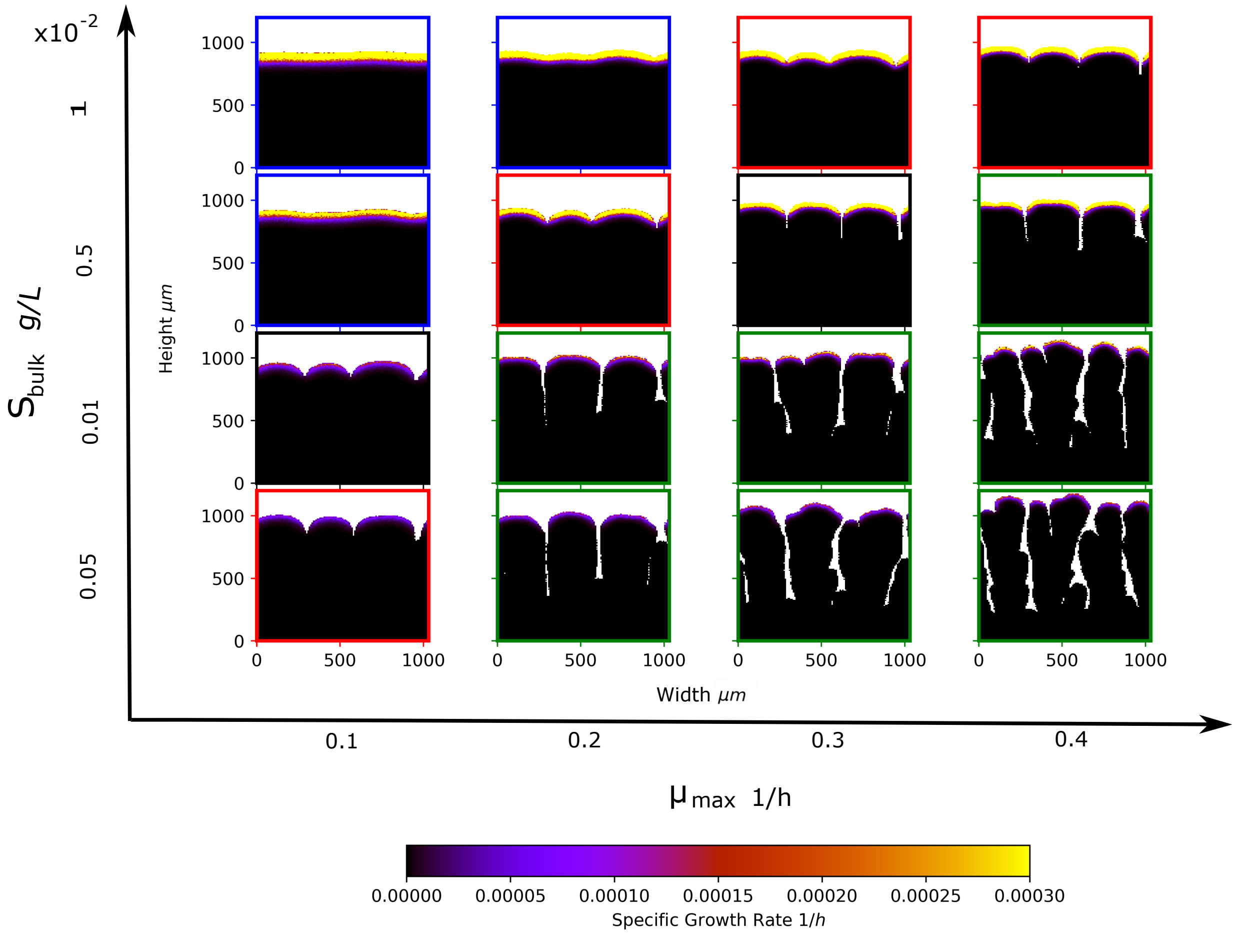

Supplementary Figure 4. Emergence of distinct biofilm morphologies. Snapshots from our grid of simulations, for biofilm sizes of approximately 75,000 cells. Our grid of simulations was defined by varying the bulk nutrient concentration S_{bulk} and maximum specific cell growth rate μ_{max}. The remainder of the simulation input parameters are held constant, and detailed in Table 1 of the main text. In the snapshots, cells are colour coded according to their specific growth rate (due to competition for nutrients this is always significantly less than μ_{max}). The coloured borders around the snapshots indicate the phase of biofilm growth - blue for unpinned, red for transiently pinned, green for pinned and black for transitional, as defined in the main text.

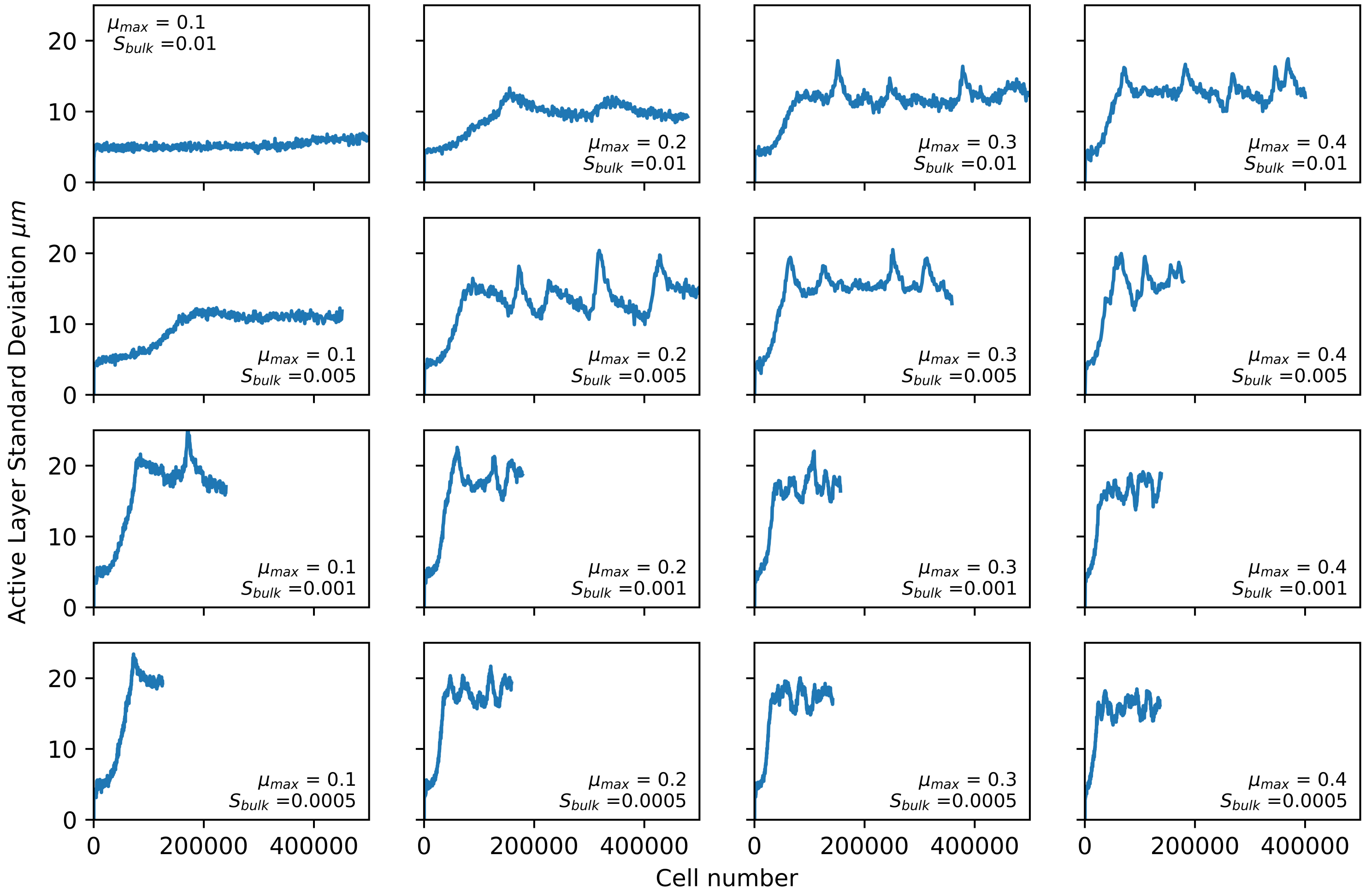

Supplementary Figure 5. Dynamics of the standard deviation of the active layer thickness. The standard deviation of the active layer thickness across the biofilm interface is plotted as a function of biofilm size for all our simulations (i.e. for simulations with different values of μ_{max} and S_{bulk}; the other input parameters are as in Table I of the main text). In the legends, the units of μ_{max} are h^{-1} and units of S_{bulk} are g/L.

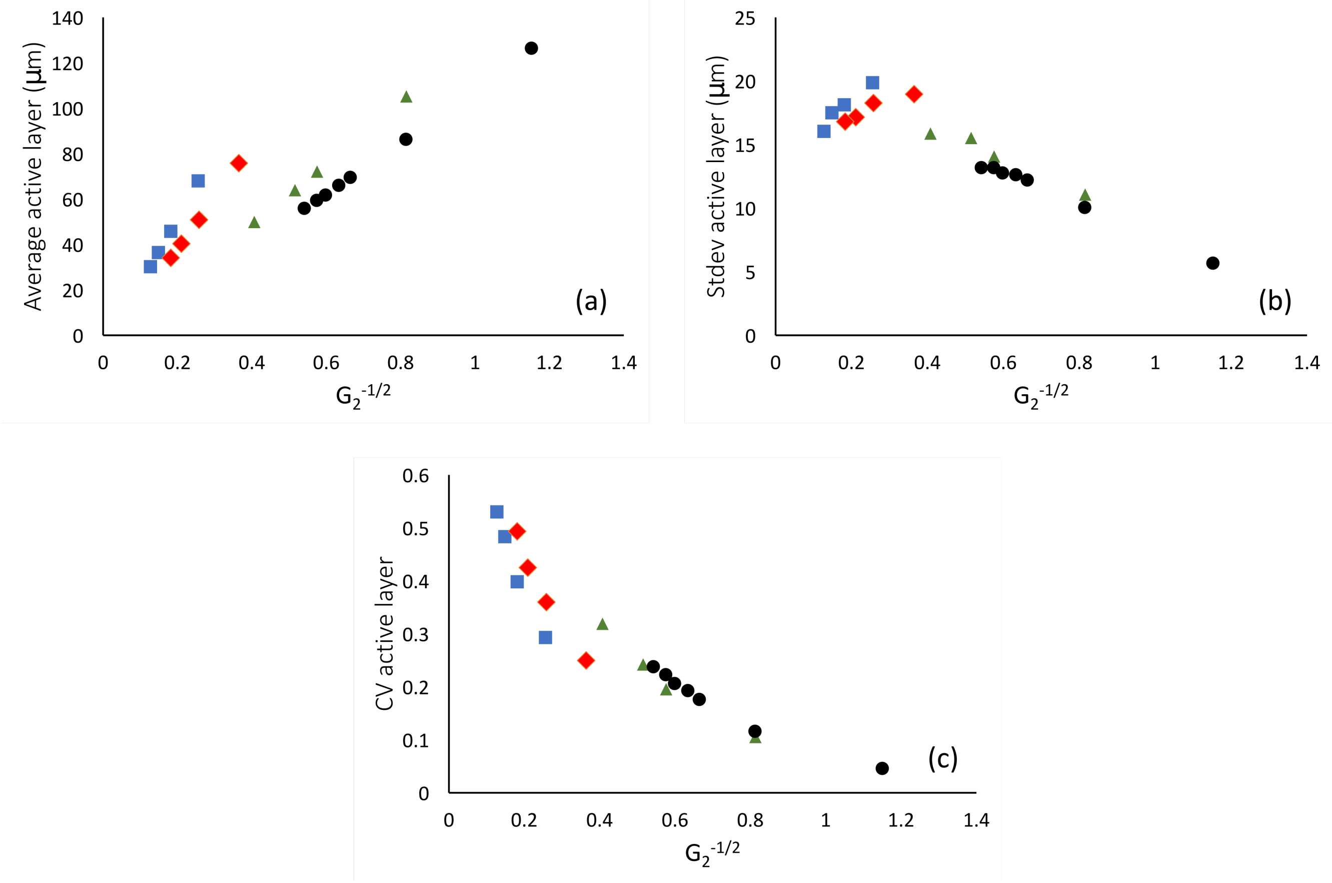

Supplementary Figure 6. Dimensionless parameter $G_2^{-\frac{1}{2}}$ and the active layer. (a): average active layer thickness (averaged across the biofilm width for steady-state simulations, see Methods), (b): standard deviation of the active layer thickness, and (c): coefficient of variation of the active layer thickness, i.e. standard deviation divided by the mean. In all panels, the parameter on the horizontal axis is the dimensionless combined parameter referred to in the main text as $G_2^{-\frac{1}{2}}$ and defined as $G_2^{-\frac{1}{2}} = (D_B Y S_{bulk})/(L_y^2 \rho \mu_{max}))^{\frac{1}{2}}$. The data points correspond to 19 simulations with different values of the parameters S_{bulk} and μ_{max}. Data points are grouped according to their S_{bulk} values. Blue squares: $S_{bulk} = 0.0005$g/L, $\mu_{max} = (0.1, 0.2, 0.3.0.4)$/h; Red diamonds: $S_{bulk} = 0.001$g/L, $\mu_{max} = (0.1, 0.2, 0.3.0.4)$/h; green triangles: $S_{bulk} = 0.005$g/L, $\mu_{max} = (0.1, 0.2, 0.25, 0.4)$/h; black circles: $S_{bulk} = 0.01$g/L, $\mu_{max} = (0.1, 0.2, 0.3, 0.33, 0.37, 0.4, 0.45)$/h.

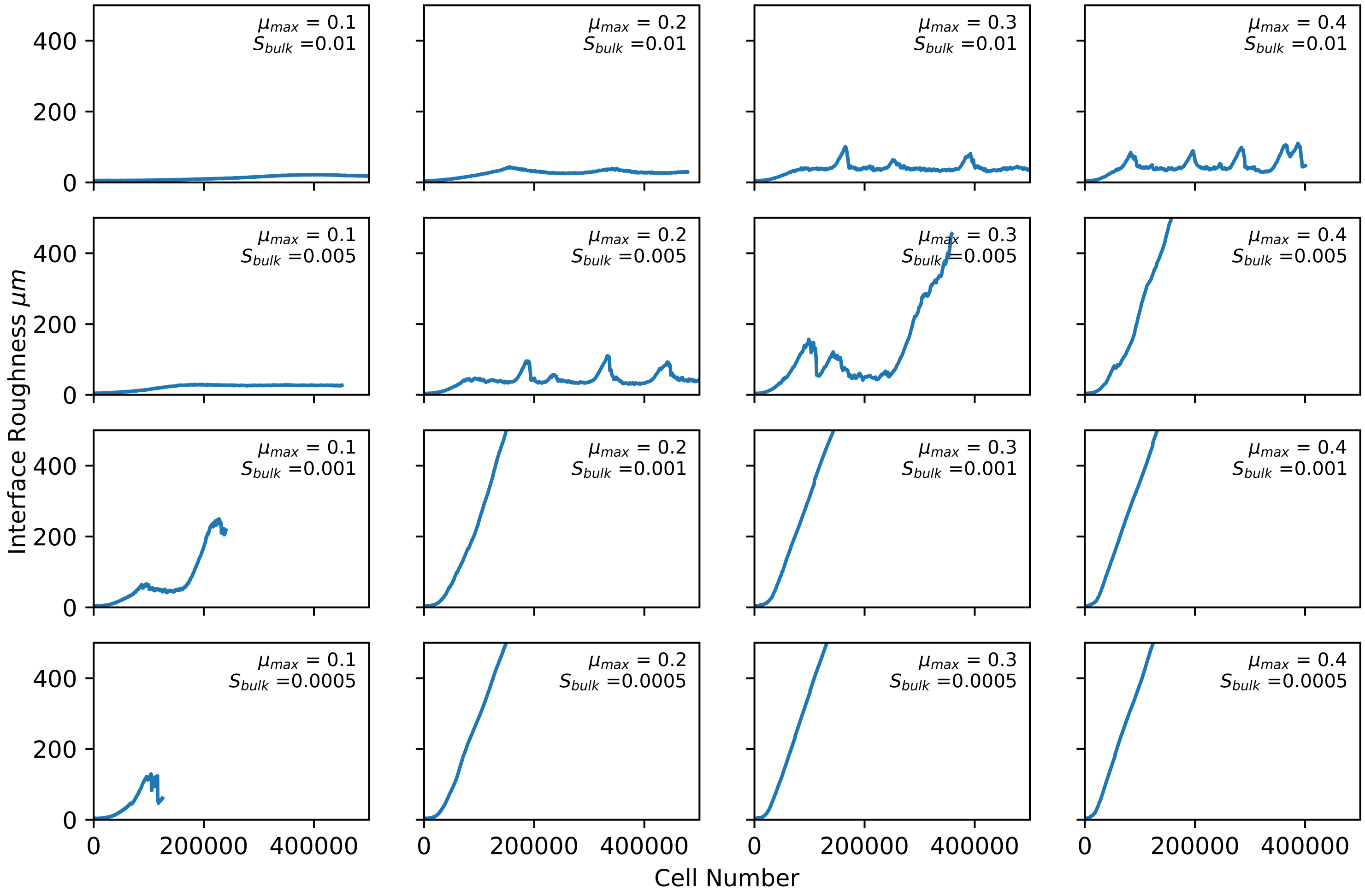

Supplementary Figure 7. Dynamics of the interface roughness. The interface roughness is plotted as a function of biofilm size for all our simulations (i.e. for simulations with different values of μ_{max} and S_{bulk}; the other input parameters are as in Table I of the main text). The units of μ_{max} are h^{-1} and the units of S_{bulk} are g/L.

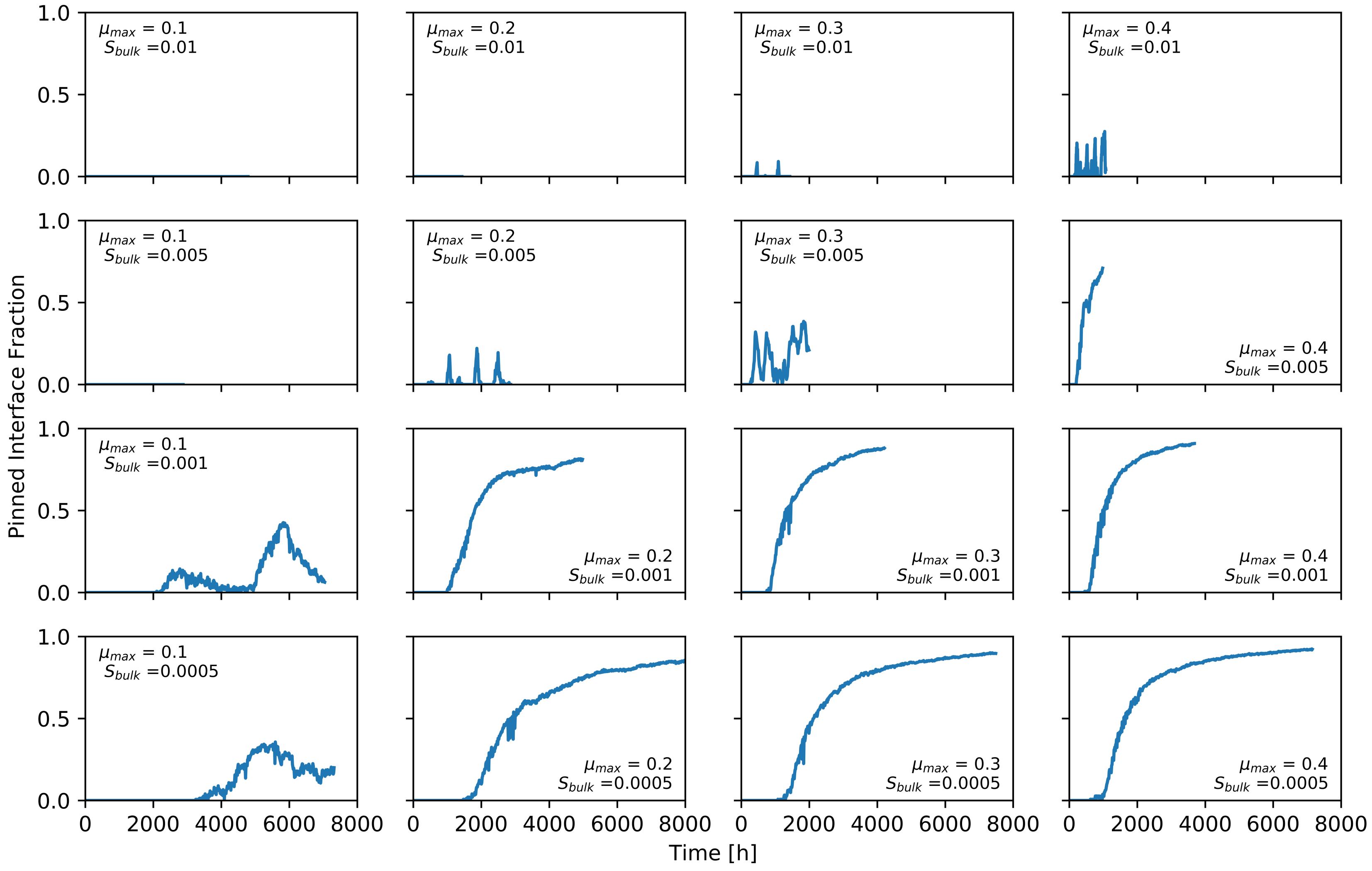

Supplementary Figure 8. Dynamics of the pinned interface fraction. The fraction of the interface that is pinned is plotted as a function of time for all our simulations (i.e. for simulations with different values of μ_{max} and S_{bulk}; the other input parameters are as in Table I of the main text). The units of μ_{max} are h^{-1} and the units of S_{bulk} are g/L. Note that, in contrast to other figures, here the trajectories are plotted against time rather than biofilm size. As discussed in the main text, simulating the pinned phase is computationally intensive. For this reason the steady state is not always reached in the pinned phase simulations.

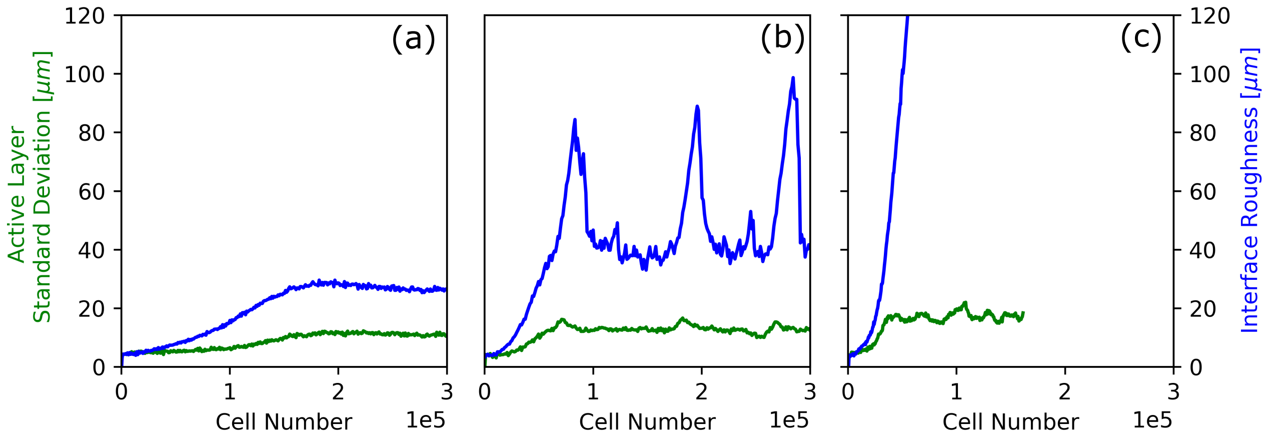

Supplementary Figure 9. Comparing the dynamics of the standard deviation of the active layer thickness and the interface roughness. The standard deviation of the active layer thickness (green lines, in μm) is plotted on the same scale as the interface roughness (blue lines, also in μm). Panel (a) shows an example simulation in the unpinned phase ($S_{bulk} = 0.01$ g/L, $\mu_{max} = 0.1$ 1/h), panel (b) shows a simulation in the transiently pinned phase ($S_{bulk} = 0.01$ g/L, $\mu_{max} = 0.4$ 1/h) and panel (c) shows a simulation in the pinned phase ($S_{bulk} = 0.0005$ g/L, $\mu_{max} = 0.4$ 1/h). The standard deviation of the active layer thickness reaches a steady state even in simulations where the interface roughness is strongly fluctuating or monotonically increasing. Comparing with Figure 5 of the main text, panels (b)-(d), we can see that when active layer gaps, and hence pinning sites, emerge, fluctuations in the active layer (standard deviation of the active layer thickness) become uncoupled from the fluctuations in the interface height (the interface roughness). In the unpinned phase (panel (a)), there is no pinning and the fluctuations in the active layer thickness and in the interface follow broadly the same trajectory. In the transiently pinned phase (panel (b)), the fluctuations follow a similar trajectory until gaps in the active layer appear (see Figure 5(c) of the main text). In the pinned phase (panel (c)), the fluctuations diverge at the point at which pinning sites arise (see Figure 5(d) of the main text).

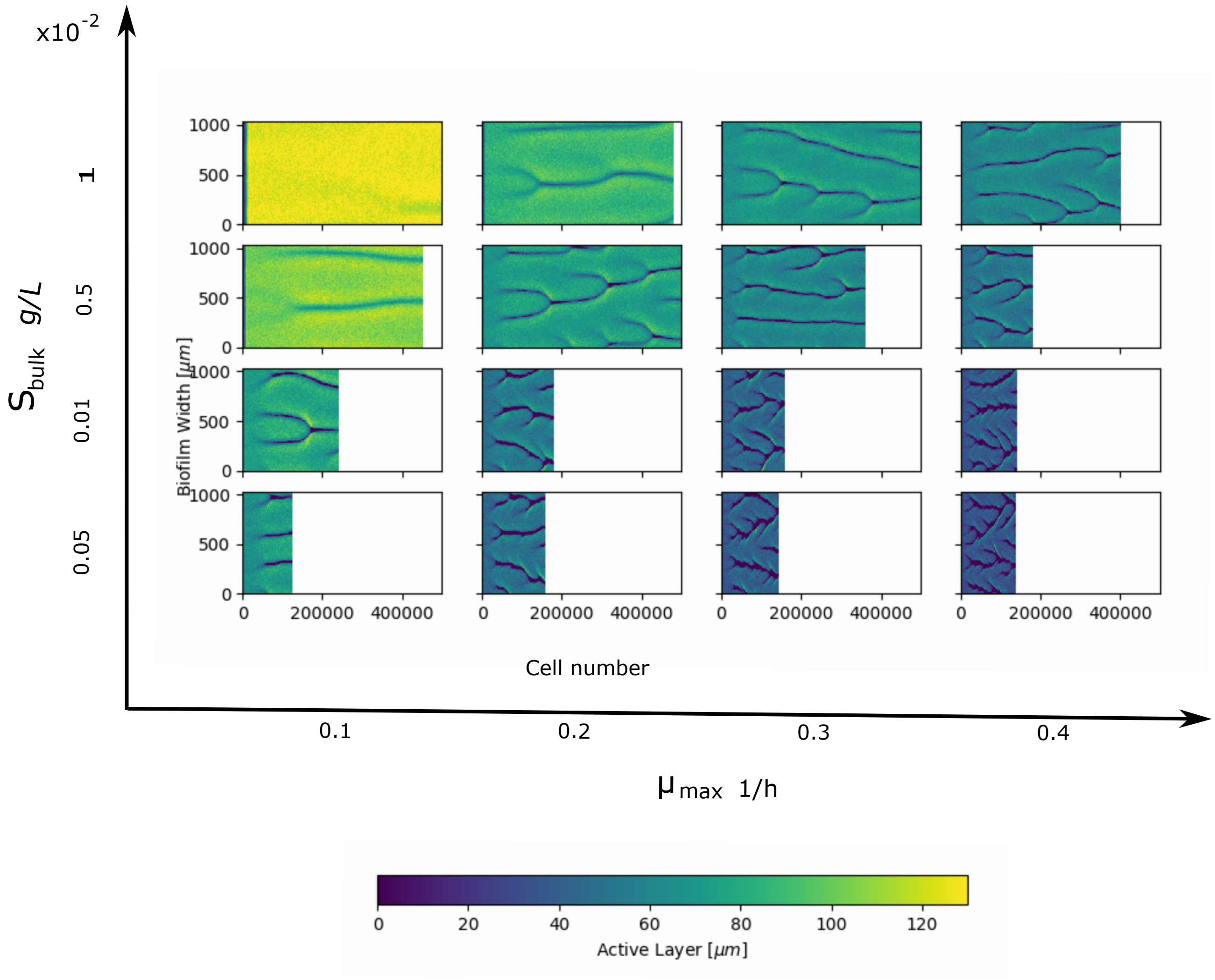

Supplementary Figure 10. Active layer gap dynamics. Kymographs of the local active layer thickness for all our simulations (i.e. for simulations with different values of μ_{max} and S_{bulk}). The other input parameters are as in Table I of the main text. For each subplot, the horizontal axis shows cell number, the vertical axis shows the position along the biofilm width and the colour shows the local active layer thickness. As in Figure 6 of the main text, dark lines on the kymographs correspond to active layer gaps. The merger of two dark lines corresponds to the engulfment of a bulge in the interface. However, it is important to note that our two dimensional biofilms have been collapsed into one dimension in these plots. This means that in the kymographs for the pinned phase (for example, $\mu_{max} = 0.3$ 1/h, $S_{bulk} = 0.001$ g/L), the apparent merging of many small active layer gaps with a larger active layer gap are in fact 'secondary' pinning sites which appear in the original biofilm fingers - or in other words, branching behaviour.

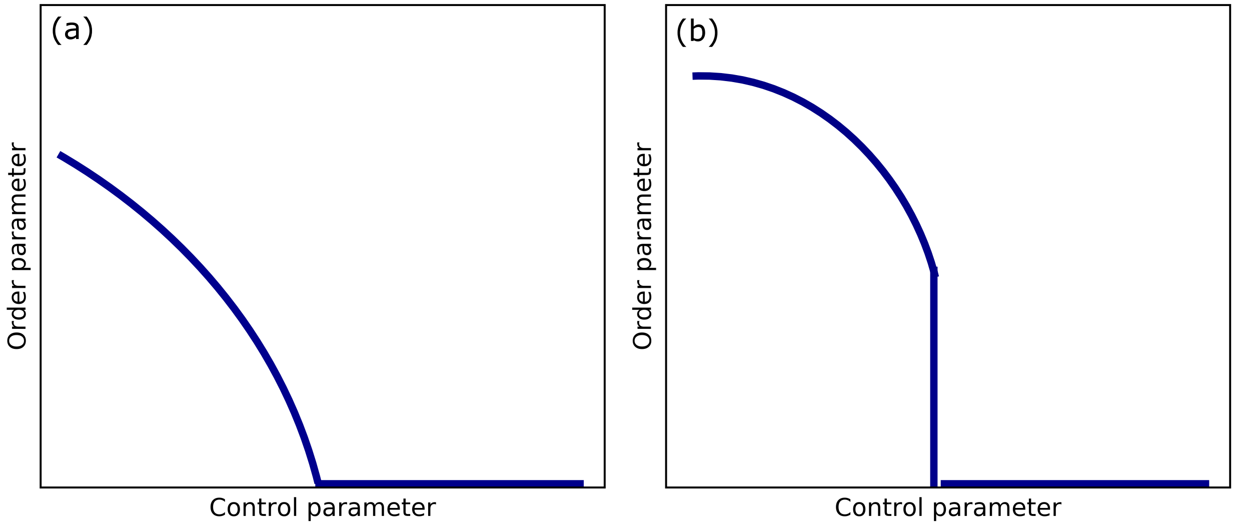

Supplementary Figure 11. **Schematic illustration of continuous and discontinuous phase transitions.** In a 'continuous transition', the order parameter changes continuously from zero to a finite value as the control parameter varies (sketch (a)). In a 'discontinuous transition', the order parameter jumps discontinuously from zero to a finite value at a critical value of the control parameter (sketch (b)).

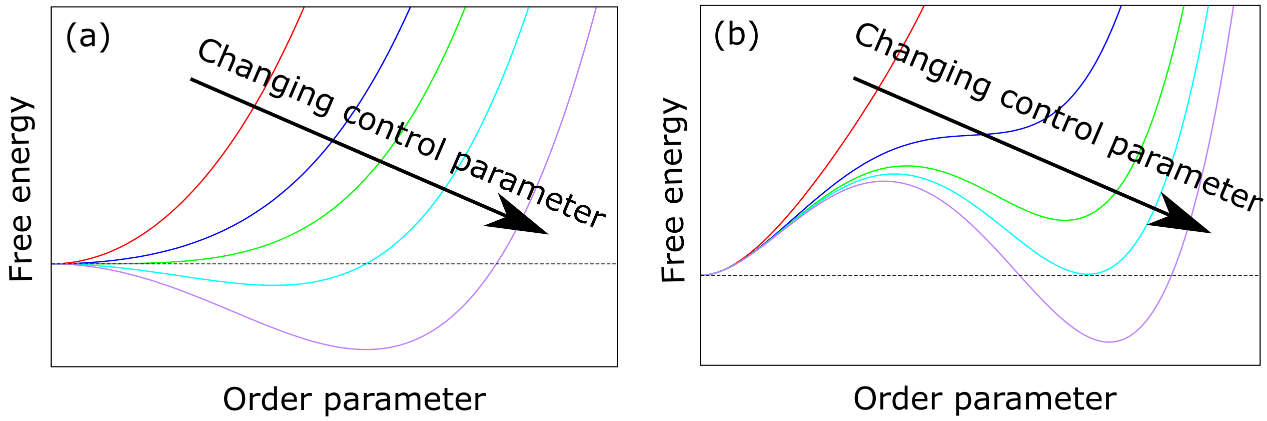

Supplementary Figure 12. Connecting continuous and discontinuous transitions to phase transition kinetics Schematic free energy profiles are sketched for continuous and discontinuous phase transitions in an equilibrium system with a non-conserved order parameter. The coloured lines correspond to different values of the control parameter. In equilibrium systems, the minimum of the free energy profile determines the equilibrium state of the system. Sketch (a) shows a continuous phase transition. Here, the free energy profile has a single minimum. Initially (red line) the minimum is at zero on the order parameter axis. Therefore the average value of the order parameter will be zero. However as the control parameter changes, the position of the minimum shifts to the right, indicating that the average value of the order parameter becomes non-zero. The position of the minimum changes continuously as the control parameter varies. A change in control parameter will result in an immediate change in system state as the free energy minimum shifts. Sketch (b) shows a discontinuous phase transition. Initially (red line), the free energy profile has a single minimum at zero on the order parameter axis; hence the average value of the order parameter will be zero. As the control parameter changes, the free energy profile develops a shoulder, and eventually a secondary minimum on the right hand side, at a non-zero value of the order parameter. This secondary minimum becomes deeper, until at a critical value of the control parameter the secondary minimum becomes the global minimum of the free energy profile. The equilibrium state of the system then shifts discontinuously from a zero value of the order parameter to the non-zero value corresponding to the secondary minimum in the free energy profile. For the discontinuous transition, because the free energy profile can have more than one minimum, there is the potential for metastability: when the control parameter changes, the system can be kinetically ‘stuck’ in a free energy minimum that is not the global minimum. To transition to the global minimum the system requires a fluctuation to overcome a free energy barrier; this phenomenon is known as nucleation. Although concepts such as free energy and metastability are ill-defined for our growing biofilms (which are far from equilibrium), nucleation-like phenomena have also been observed in non-equilibrium systems.

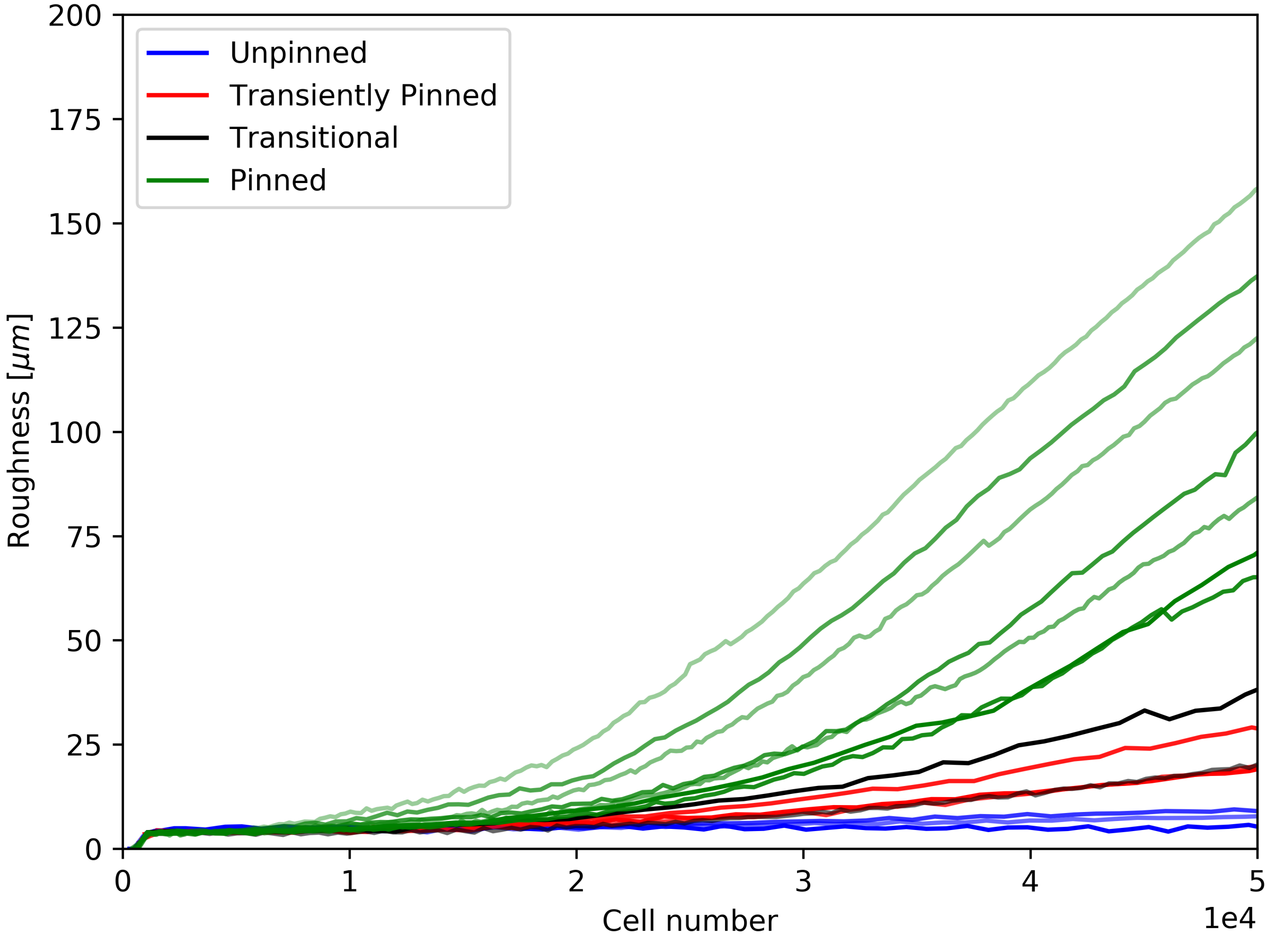

Supplementary Figure 13. Interface roughness at early times. Early-time dynamics corresponding to the interface roughness trajectories of Figure 6(a) of the main text. In most of the simulations, there is a 'pseudo-steady state' during the early growth behaviour (i.e. for small cell numbers $< 1 \times 10^4$), before the roughness further increases. The behaviour at these early times is very different to the true steady-state behaviour that we observe at later times.

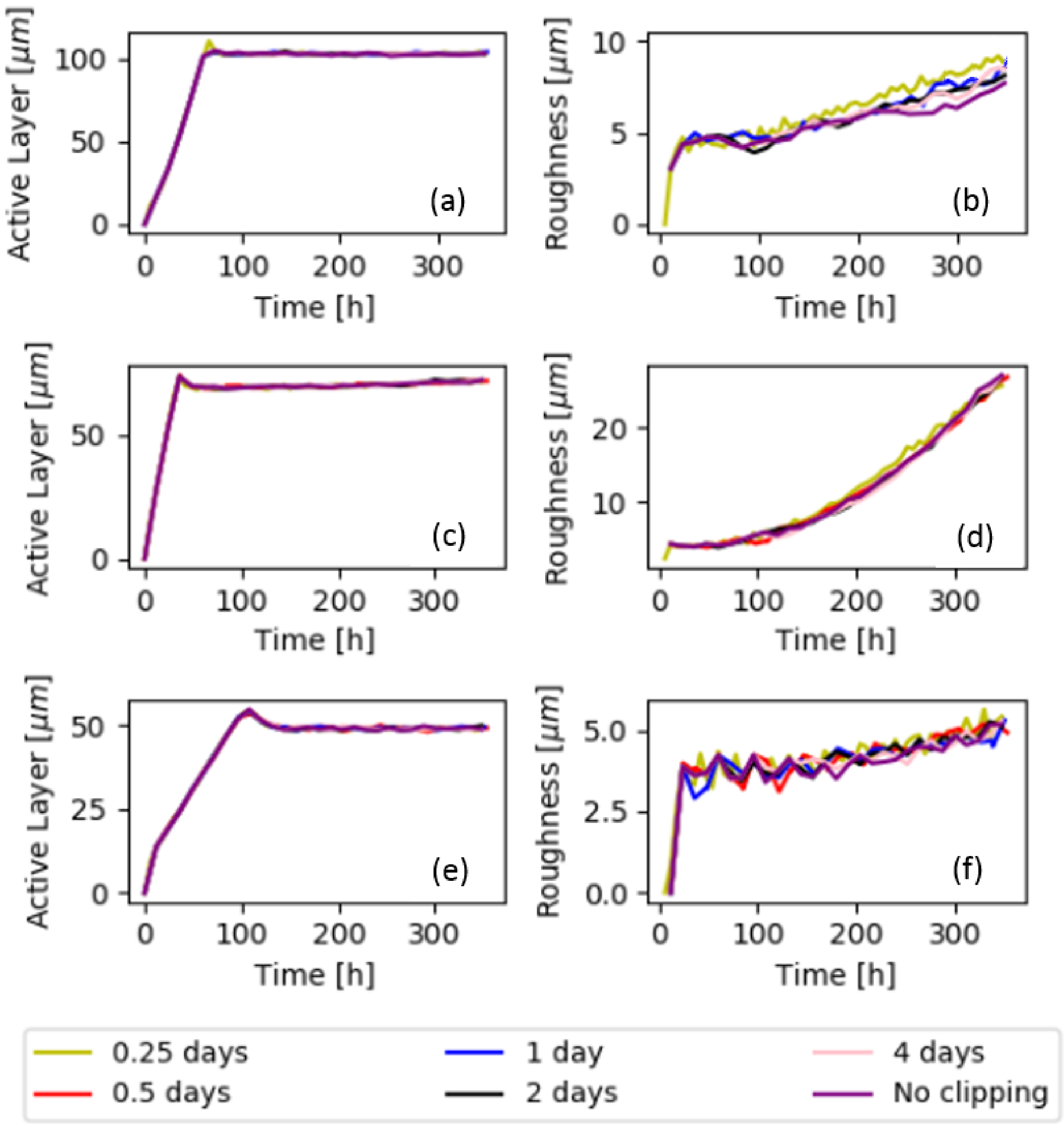

Supplementary Figure 14. Testing the clipping algorithm with short simulations. The results of clipped simulations are compared to those for a simulation without clipping. This comparison is only possible for short simulations; for longer times the unclipped simulations become too computationally demanding. We plot trajectories of the average active layer thickness and interface roughness for test simulations with different frequencies of clipping and without clipping. Each pair of plots (a) and (b); (c) and (d); (e) and (f) are for sets of simulations with different parameter sets. The different coloured lines on these plots are for simulations with a different simulation segment length T_s (i.e. a different clipping frequency; indicated in the legend) and for a continuous simulation in a single segment (i.e. no clipping). The results show that the trajectories of the active layer thickness and the interface roughness do not depend on the clipping frequency. We note that iDynoMiCS simulations are inherently stochastic, so trajectories are not expected to be exactly identical.

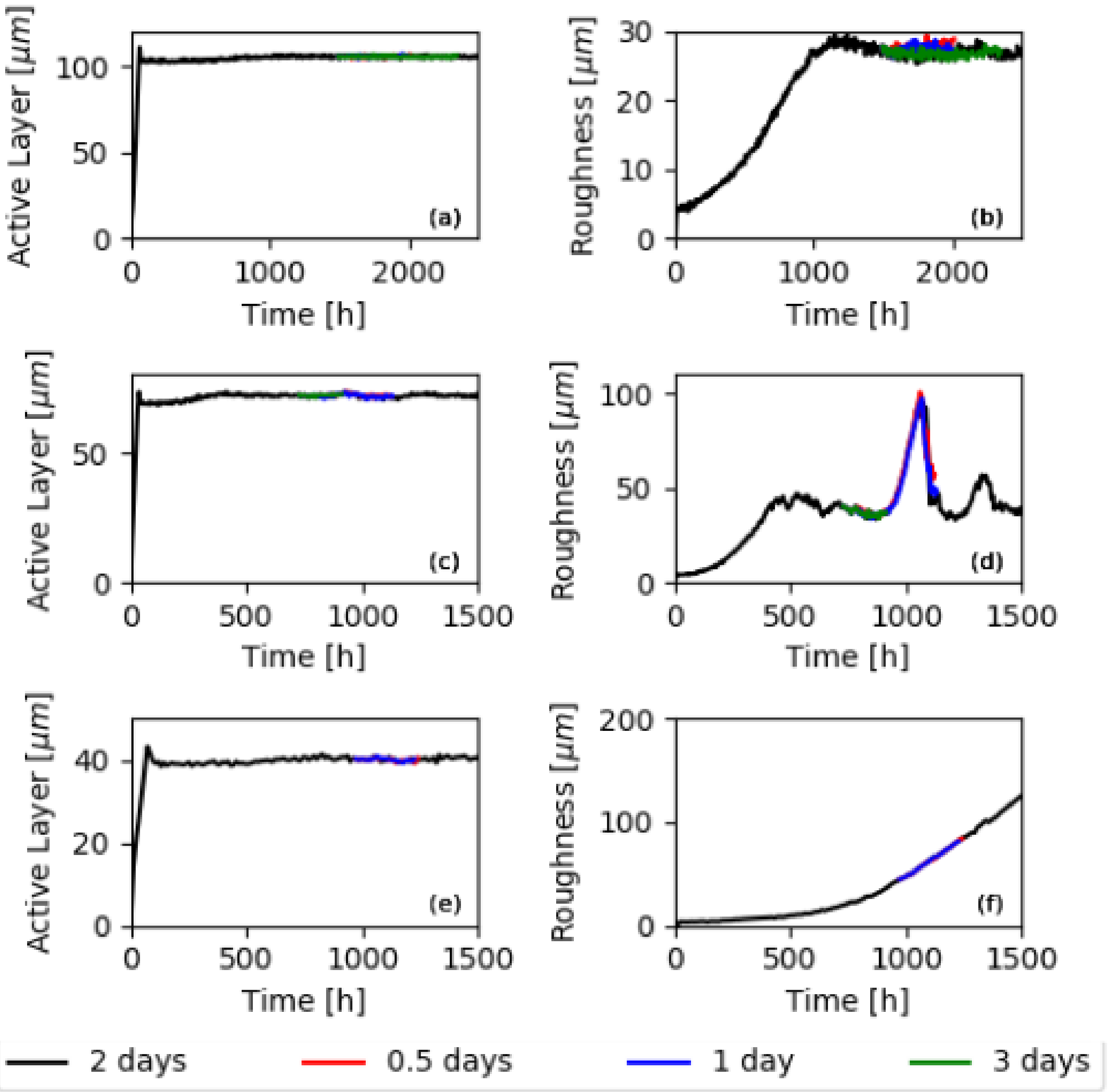

Supplementary Figure 15. Testing the clipping algorithm with long simulations. Results are compared for long time simulations with different clipping frequencies. We plot trajectories of the average active layer thickness and interface roughness for test simulations with different frequencies of clipping. Each pair of plots (a) and (b); (c) and (d); (e) and (f) are for sets of simulations with different parameter sets. The different coloured lines on these plots are for simulations with a different simulation segment length T_s (i.e. a different clipping frequency; see legend). The black line shows results from a long simulation with clipping frequency once per 2 days; new simulations with different clipping frequencies were initiated from a mid-time configuration of the black simulation. The results show that the simulation trajectories do not depend on the clipping frequency; hence we conclude that the clipping procedure does not significantly perturb the simulations.

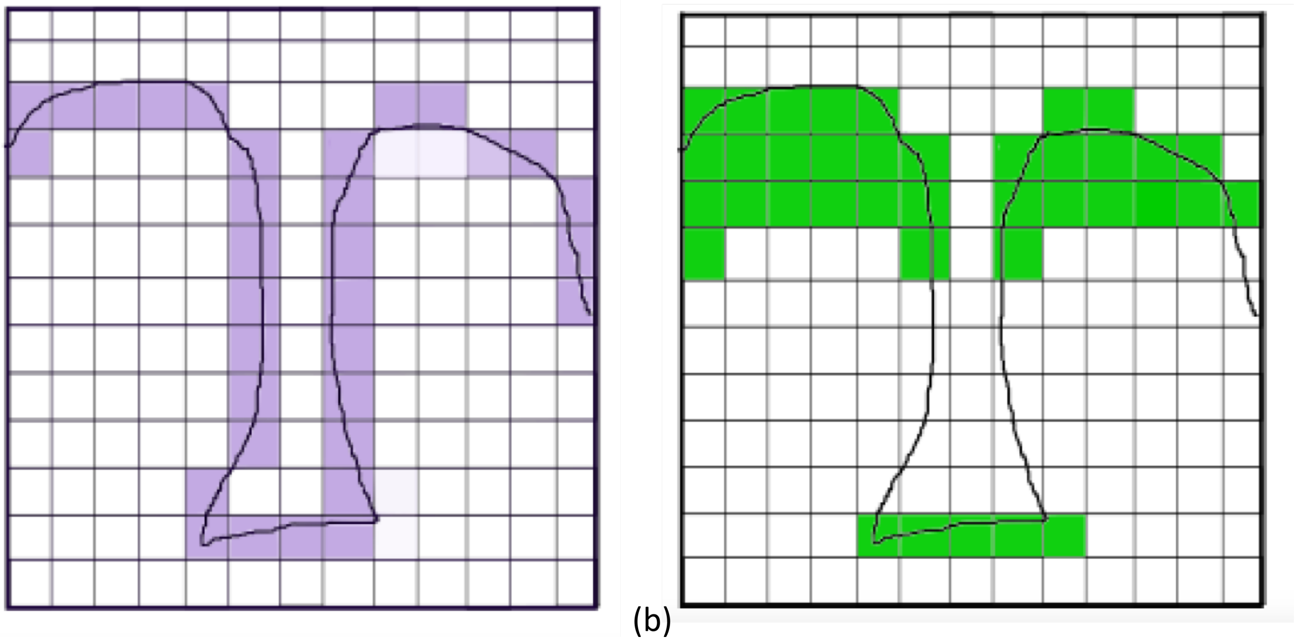

(a) (b)

Supplementary Figure 16. Diagrams illustrating the calculation of interface roughness and the definition of the active layer (see also the Methods section of the main text). (a): The roughness is calculated as the standard deviation of the vertical height of the purple shaded grid squares, i.e. all grid squares that contain biomass and are adjacent to a square that does not contain biomass. (b): The active layer is defined to include the green shaded grid squares. These are all squares where the local growth rate exceeds the active layer threshold; note that they can, in principle, be located at the base of troughs in the interface (although Figure 2 in the main text shows that this is not usually the case). The active layer thickness at a particular horizontal position is calculated from the number of shaded grid squares in the corresponding vertical column.
